# Using Magnesium and Magnesium-Based Alloys as a Novel Biomaterial to Create Medical Devices by AM Techniques—A Review

**DOI:** 10.3390/ma19132890

**Published:** 2026-07-06

**Authors:** Corneliu Munteanu, Ioana-Ilinca Volocaru, Boris Nazar, Fabian-Cezar Lupu, Bogdan Oprisan, Ioana-Alexandra Stan, Grigorii Deleu, Gabriela Stan

**Affiliations:** 1 Faculty of Mechanical Engineering, “Gheorghe Asachi” Technical University of Iasi, 63 D Mangeron Blvd, 700050 Iasi, Romania; corneliu.munteanu@academic.tuiasi.ro (C.M.); fabian-cezar.lupu@academic.tuiasi.ro (F.-C.L.); ioana-alexandra.stan@student.tuiasi.ro (I.-A.S.); grigorii.deleu@student.tuiasi.ro (G.D.); gabriela.stan@student.tuiasi.ro (G.S.); 2 Technical Science Academy Romania, 26 Dacia Blvd, 030167 Bucharest, Romania; 3 Department of Manufacturing Engineering, Technical University of Moldova, Bd. Stefan cel Mare 168.1, MD-2004 Chisinau, Moldova; boris.nazar@if.utm.md; 4 Department of Manufacturing Engineering, University of Medicine and Pharmacy “Grigore T. Popa” of Iasi, Str. Universitatii 16, 700115 Iasi, Romania

**Keywords:** magnesium, AM, biocompatibility, 3D printing, scaffolds

## Abstract

Magnesium alloys are considered to be the third generation of biomaterials used in biomedical applications to promote bone tissue regeneration. Due to their Young’s modulus being similar to that of human bone and their release of magnesium ions that are antimicrobial and osteoinductive, these biomaterials not only promote bone regeneration, minimize the effects of stress shielding and reduce the risk of infection, but also their exceptional biocompatibility and bioresorbability eliminate the need for a second surgery to remove the implant. However, because magnesium has poor corrosion resistance, without different coatings and surface treatments, the implant can be compromised before the bone is fully healed. With additive manufacturing (AM) as a revolutionary technology, the one-size-fits-all approach can be replaced by fully personalized medicine, in which complex shapes can be created, designed, and processed with unique parameters for each patient. However, 3D printing of Mg-based devices remains particularly challenging due to magnesium’s high chemical reactivity, combustion risk, and low vaporization temperature, challenges that are further compounded when alloying elements are introduced. This review addresses this gap by critically examining the properties, corrosion behavior, and bio-medical performance of Mg and its alloys, with a focused analysis of selective laser melting (SLM) and wire arc additive manufacturing (WAAM) as key fabrication methods. The influence of processing parameters, microstructural defects, and alloy composition on the final properties of AM-fabricated Mg components is systematically discussed, alongside current limitations and prospective strategies toward their clinical translation.

## 1. Introduction

As a general rule, a successful biodegradable implant must fulfill three main requirements: providing enough mechanical support during the healing process, complete degradation when the tissue is fully regenerated, and enhancing complete replacement by the newly formed tissue [1,2]. The healing process of regeneration and repair of bones after injury is a complex mechanism [3,4]. This process is divided into three phases: hematoma mechanization—0–2 weeks; bone scab formation—6–8 weeks; and bone scab remodeling and shaping phase—8–12 weeks [5,6].

Magnesium and its alloys are biodegradable materials with a wide range of applications, including orthopedics, dentistry, and cardiovascular devices [3,7,8,9,10,11]. In comparison with other metallic implant materials, magnesium is biodegradable, and during the healing process, magnesium continues to degrade, thereby eliminating the need for a second surgical intervention. Mg and its alloys offer mechanical properties similar to those of human bone, which can prevent stress shielding [12,13,14]. Furthermore, the magnesium ions released during degradation are osteoconductive and antimicrobial, enhancing new bone formation and reducing the risk of infection. One of the main drawbacks of magnesium is its rapid corrosion rate in the physiological environment. Therefore, facilitating an ideal result where the corrosion rate and the degradation process match the healing time of bone tissue remains a challenge [15,16]. Heat treatment is considered one of the most effective strategies to improve both the corrosion rate and the mechanical properties of Mg alloys without compromising their composition or geometry [17,18,19]. The specific methods (T4, T5, and T6) and their effects are discussed in detail in Section 5.1 [20]. For different types of Mg alloys fabricated via additive manufacturing techniques, e.g., AZ80M, AZ91D, and WE43, the effect of heat treatment was studied, with results showing significant improvements in microstructure, mechanical properties, and corrosion resistance compared to samples that did not undergo post-processing treatment [10,21,22,23].

Additive manufacturing (AM) offers a completely new perspective in guided bone regeneration (GBR) through its capability to create complex and fully personalized devices tailored to meet each patient’s specific needs. With the aid of 3D printing techniques, the paradigm of one size fits all shifts toward personalized medicine, with a focus on creating devices that are specifically designed and manufactured to address each individual clinical case [24,25]. Conventional techniques deliver limited options, where features such as customization, microstructure or porosity often cannot be tailored, both representing critical aspects in bone healing and integration [7,26,27]. Among AM methods, SLM and WAAM are particularly suited for Mg alloys and are discussed in detail in Section 3.

However, 3D printing using magnesium alloys is a particularly challenging process due to the high chemical reactivity of magnesium, which can lead to combustion risk [27]. Several key methods can be employed to minimize and control the flammability of magnesium, including microstructure management through the reduction of low-melting-point eutectic phases via heat treatment; the formation of protective surface films through the addition of specific alloying elements that generate a dense, compact oxide layer; and alloying with rare earth elements (REE). The addition of yttrium (Y), gadolinium (Gd), and neodymium (Nd) significantly increases the ignition temperature through the formation of a protective surface film. A 5 wt.% addition of Y, Gd, or Nd has been reported to increase ignition resistance during flame testing. Calcium (Ca) additions of up to 3 wt.% improve oxidation resistance, while beryllium (Be) is highly effective in reducing oxidation during melting [28]. Control of environmental conditions through strict handling protocols and the use of shielding gas atmospheres, such as SF_6_, N_2_, or inert gas covers, is also employed to prevent ignition [29].

This work reviews magnesium and magnesium alloys from two complementary perspectives. First, it provides a general overview of the fundamental properties and characteristics of magnesium. Second, it presents an in-depth analysis of magnesium and its alloys produced through additive manufacturing techniques, particularly selective laser melting (SLM) and wire arc additive manufacturing (WAAM). The review discusses the fabrication of magnesium-based materials using SLM and WAAM, with emphasis on the influence of processing parameters on microstructural evolution, mechanical performance, and defect formation. In addition, a brief overview of the potential applications of additively manufactured magnesium alloys is provided.

## 2. Properties of Additively Manufactured Mg and Mg Alloys

### 2.1. Mechanical Properties

Medical implants should be able to support anatomic loads [30]. Therefore, mechanical properties such as ductility, hardness, compressive and tensile strength, elastic modulus and toughness require thorough investigation prior to clinical application. A discrepancy between the elastic modulus of the implant and that of the surrounding tissue can cause stress shielding, a phenomenon that occurs when the implant bears mechanical load instead the bone, potentially leading to bone resorption and implant failure.

Pure Mg generally exhibits low tensile strength and medium ductility, which renders it insufficient as a standalone material for applications demanding high mechanical performance. For this reason, alloying is widely employed as an effective strategy to enhance the mechanical properties of Mg, making it a more suitable candidate across a broad range of biomedical applications. The most commonly used non-rare earth element (non-REE) and rare earth element (REE) alloying additions include: Al, Ca, Zn, Mn, Y, Nd, Gd and La. Together with Mg, these alloys can be processed via AM techniques such as SLM, WAAM, binder jetting, extrusion-based deposition to fabricate a wide range of patient-specific medical devices, including orthopedic screws, scaffolds, stents, and surgical staples, across numerous medical fields [31,32,33]. Table 1 highlights and summarizes the mechanical properties of several SLMed and WAAMed Mg alloys [34,35]. Alloying Mg with non-REE and REE elements significantly enhances its mechanical and corrosion performance [36]. For instance, Mg-Nd and Mg-Er, alloys are noteworthy for their elevated high-temperature mechanical properties. However, their corrosion resistance is not an intrinsic characteristic but rather a context-dependent outcome strongly influenced by alloy microstructure, second-phase distribution, grain boundary chemistry, and the specific AM processing parameters employed [37,38,39,40]. For Mg-Ca alloy, the mechanical properties are strongly dependent on Ca content; specifically, the addition of 1 wt.% Ca has been reported to yield the most thermodynamically stable inter-metallic phase, resulting in optimal mechanical performance [41,42]; However, increasing the content of Ca can lead to negative effects on the tensile properties.

For SLM-processed Mg alloys, mechanical properties are strongly governed by grain size, solute concentration and second-phase distribution, microstructural features that are directly influenced by the applied volumetric energy density. Optimizing the energy density promotes grain refinement, solid solution strengthening, and precipitate strengthening, collectively resulting in improved yield strength and ultimate tensile strength. Pure Mg processed by SLM has been reported to reach hardness values of 60–89 HV [43], demonstrating the capability of this AM technique to enhance the hardness of Mg-based components. Furthermore, SLM-processed Mg devices exhibit lower fracture elongation compared to conventionally manufactured counterparts, typically below 10% [44,45,47], a limitation attributable to a combination of factors, including elevated residual stress levels, formation of intermetallic compounds along grain boundaries, process-induced porosity arising from gas entrapment or incomplete powder melting, crystallographic anisotropy resulting from the preferential columnar grain growth along the building direction, and lack-of-fusion defects caused by insufficient overlap between successive melt pools or scan tracks [72]. In contrast to SLM, which can produce devices with high surface quality and high yield strength but involves inherent safety hazards associated with the use of reactive Mg powder as feedstock, WAAM utilizes wire-based raw material, thereby significantly reducing combustion and explosion risks. The mechanical properties and overall quality of WAAM-processed Mg alloys are strongly dependent on welding parameters and torch feed speed, which must be carefully optimized to minimize porosity, thermal distortion, and microstructural anisotropy.

For both SLM and WAAM mechanical properties that meet the requirements of the intended clinical application, careful selection of alloying elements and process input parameters is essential. Furthermore, post-processing operations, discussed in detail in a subsequent chapter, play an essential role in enhancing the overall quality and performance of AM-fabricated medical devices.

### 2.2. Corrosion

Corrosion rate is a key factor in determining the performance of a biodegradable implant under physiological conditions. There are two main methods used to evaluate the corrosion rate: in vivo and in vitro. In vitro testing refers to replicating the physiological environment outside a living organism, either through immersion tests, in which the medical device is submerged in a simulated body fluid (SBF), or through electrochemical testing, in which electrical techniques are used to determine the corrosion behavior [36]. In vivo testing, on the other hand, requires the direct implantation of the device in a living organism, followed by measurement of mass loss over a defined period of time. In order to obtain valid and comprehensive results, both methods should be employed, as neither in vitro nor in vivo testing alone accurately captures the full corrosion behavior of Mg and its alloys. Significant differences between in vivo and in vitro corrosion behavior of Mg-based alloys have been widely reported, with in vitro conditions generally yielding higher corrosion rates, influenced by factors such as immersion medium composition, pH stability, absence of serum proteins, and static rather than dynamic flow conditions, none of which fully replicate the complex physiological environment in vivo. A notable exception to this trend has been reported for Mg-6Zn alloys [36,73,74]. Current density, potential, and electrochemical impedance are key electro-chemical parameters governing the corrosion behavior of Mg alloys; materials exhibiting a more positive corrosion potential generally demonstrate higher corrosion resistance [75,76]. The corrosion process typically initiates at surfaces and edges before propagating into the bulk material and affecting the microstructure. Surface treatment represents an effective strategy to improve corrosion resistance and thereby extend the functional lifetime of the implanted device [77,78].

This section highlights and summarizes the influence of both REE and non-REE alloying elements on the corrosion resistance of magnesium. Although the Mg-Al system is one of the most widely used alloy families, aluminum offers effective solid solution and precipitation strengthening in magnesium [79,80,81,82]. In Mg-Al alloys, Al promotes the formation of the intermetallic phase Mg_17_Al_12_, which is typically distributed along grain boundaries or dissolved within the magnesium matrix, depending on the processing and heat treatment conditions. The effect of Mg_17_Al_12_ on corrosion resistance is highly dependent on its morphology and distribution: a fine and homogeneous dispersion of this phase can contribute to improved corrosion resistance by reducing micro-galvanic coupling and promoting the formation of a more stable protective surface film. However, a coarse or continuous distribution along grain boundaries can accelerate localized corrosion through micro-galvanic effects [83,84]. Magnesium and its alloys are highly sensitive to impurities, which represent one of the primary sources of corrosion [85]. The addition of manganese (Mn) to magnesium has been shown to effectively reduce impurity levels through the formation of new intermetallic phases with iron (Fe) and other heavy metal elements, thereby improving corrosion resistance [86,87]. Rare earth elements (REE) enhance the microstructure and mechanical properties of Mg alloys and also improve their corrosion resistance, attributed to their unique extranuclear electron configuration [88,89]. For Mg-xNd alloy (x = 0, 0.5, 2, 5 wt%), Zhang et al. [36,90] studied the degradation process in an SBF. An initial increase in corrosion rate was reported, followed by subsequent reduction, with this trend being most pronounced at a Nd content of 2 wt.%. This behavior can be attributed to the formation of Mg_41_Nd_5_ intermetallic particles at higher Nd concentrations, which promote micro-galvanic corrosion. The presence of yttrium (Y) in magnesium alloys promotes the refinement of α-Mg grains and purifies the Mg melt, leading to a reduction in the intensity of galvanic corrosion. Furthermore, for Mg-Zn-Ca-Y alloys, corrosion resistance was observed to improve continuously with increasing Y content, as evaluated in Hank’s Balanced Salt Solution [91].

Due to the rapid solidification characteristic of SLM, the microstructure is significantly refined and second phases are largely dissolved into the matrix. Furthermore, the dominant corrosion mechanism can shift from pitting corrosion, typical of conventionally processed Mg-Al alloys, to a more uniform overall corrosion [92]. In a study performed by Izumi et al. [93], the cooling rate has a major influence on the corrosion behavior of magnesium alloys, thus increasing the cooling rate delays, the appearance of filamentous corrosion due to grain refinement. Table 2 highlights the corrosion resistance of SLMed Mg alloys [94].

For WAAMed Mg alloys, corrosion resistance is strongly microstructure-dependent. The strategies for its optimization through process parameter control and heat treatment are discussed in detail in Section 3.2. Table 3 shows the corrosion properties for some WAAMed Mg alloys. 

Both SLM and WAAM improve the corrosion resistance of Mg alloys through rapid solidification and refined microstructures, making careful process parameter selection essential for meeting specific implant performance requirements.

### 2.3. Biocompatibility

Biocompatibility and non-toxicity are the primary and most critical requirements for any implant material. Following implantation into the human body, biological reactions are initiated between the implanted device and the host tissue, and it is this interaction that determines whether the medical device is accepted or rejected [105]. Temporary implants function as mechanical support for the fractured bone and must maintain this function until the injury is fully healed, after which the degradation process should occur within the body at a controlled rate [106,107]. Upon interaction with the physiological environment, temporary implants must be non-toxic and must not cause any adverse biological reaction. Magnesium is naturally present in bone tissue and its high biocompatibility makes magnesium alloys an excellent candidate for temporary implant applications. Approximately 21 to 35 g of magnesium can be stored in the body of a healthy adult [108,109], of which approximately 20% is found in bone, 35–40% is sorted into tissue and ligaments, and 1% is present in body fluids [110]. The recommended daily intake of Mg is approximately 420 mg, making magnesium the second most consumed essential nutritional element. Furthermore, the degradation products generated during the corrosion process can be absorbed or excreted through urine without causing any harm to the body [111,112]. In the case of Mg-Al alloys, given that aluminum is a neurotoxic element, studies show that Al content exceeding 5 wt % can cause damage to neurons and osteoblast cells. It is therefore essential to consider regulatory guidelines and biocompatibility requirements prior to the clinical implantation of any medical device [113]. For RE-Mg alloys, results are very promising. In combination with magnesium, these alloys deliver excellent cell survival rates, with cell viability exceeding 100% after one day of culture [114]. Moreover, certain rare earth elements exhibit higher cell viability compared with pure Mg, suggesting that the risk of cytotoxicity associated with their addition is not significant. Mg-Nd alloys in combination with Zn, and Zr have been shown to further enhance cell proliferation and differentiation. However, in the case of Yb and Tm, it is assumed that some cytotoxic effects may occur due to the low cell viability that has been observed. This limitation can be mitigated by adjusting the concertation of Yb and Tm or by developing multi-element RE systems that achieve lower cytotoxicity while preserving their advantageous biological properties.

### 2.4. Osteogenesis and Angiogenesis

Osteogenesis is defined as the biological process by which new bone tissue forms in order to repair a fractured bone. An important consideration is that, during the healing of a fractured bone, osseous union should be achieved before the temporary implant fully degrades in order to ensure a satisfactory clinical outcome. As a physiological process, osteogenesis is highly influenced by cellular, biochemical and pathological factors. Magnesium and its alloys possess inherent osteogenic properties, making them more advantageous for temporary implants compared to bioinert materials. Feng et al. [115] investigated the osteogenic performance together with the degradation behavior and antimicrobial properties of a ZK60 Mg alloy coated with Ga. They used Ga as a coating with the aim to improve the degradation rate and ensure the mechanical strength of the implant, as well as to promote osteogenesis and inhibit osteoclast in order to maintain bone stability [115,116]. After coating MgCa1 alloy with Zn and Ga, it was observed that the number of cells attached to Ga (6.3 × 10^4^ cells/cm^2^) was significantly higher in comparison with Zn (2.6 × 10^4^ cells/cm^2^) [117]. The obtained results show that co-culturing with human bone marrow-derived mesenchymal stem cells (hBMSCs) in alloy extracts revealed that a longer alloying time for Mg-Ga led to an enhancement of the spreading, proliferation and differentiation of these cells. Moreover Ga, inhibited the osteoclast differentiation, and the released ions of Mg, Zn and Ga promoted the osteogenesis performance overall.

Rear earth-Mg alloys are another category that stands out for their capability to promote bone formation. RE ions are able to stimulate the sensory nerve ending, which leads to a release of neurotransmitters, a process that ultimately enhances the differentiation of stem cells towards an osteogenic lineage [118,119]. Due to their chemical similarity to calcium (Ca), RE ions represent promising candidates for promoting bone regeneration and the treatment of bone diseases. The osteogenic effects of RE-Mg alloys can be attributed to two mechanisms: the first is pro-osteogenesis, whereby bone cell proliferation and differentiation are stimulated through the activation of relevant signaling pathways [36]. For instance, lanthanum (La) is able to activate Wnt/β-catenin or TGF-β pathways, ultimately inducing elevated expression of osteogenic genes responsible for bone formation [120]. Even though in comparison with RE-Mg alloys, ceramics can provide and improve stem cell proliferation and enhance bone regeneration, they show some limitations due to their brittleness and high Young’s modulus. In contrast, RE elements are a remarkable choice for temporary implants. They offer excellent biocompatibility and capacity to enhance osteogenesis together with Mg. Additionally, studies show that when adding RE elements, mechanical properties and processing characteristics are substantial improved.

For successful tissue regeneration, early formation of a sturdy network of blood vessels, a phenomenon known as angiogenesis, is mandatory. The newly formed network of vessels is a key factor in delivering nutrients, growth factors, and oxygen. Without this process, supporting cellular activity and the growth of new tissue would not be possible [121]. Mg alloys, in a recent study, showed great ability to positively influence angiogenesis. For Mg-Li-Al-RE alloys, a high survival rate of human umbilical vein endothelial cells (HUVECs) was observed [122]. Therefore, managing to control the degradation behavior of Mg alloys represents a promising starting point in enhancing endothelization and angiogenesis performance.

## 3. Additive Manufacturing of Magnesium Alloys

Three-dimensional (3D) printing technology is becoming increasingly relevant in the biomedical field. Together with biomaterials and tissue engineering, it has gained popularity as a preferred alternative in the healing of bones, teeth or organs. The overall purpose of creating temporary devices designed and fabricated via additive manufacturing using biomaterials is to eliminate the need for a second removal surgery and to create specific shapes tailored to the various requirements of each patient [123]. Table 4 summarizes the advantages, disadvantages and applications of different 3D printing techniques [36,124]. This paper will focus on SLM and WAAM.

Compared to other AM processes, devices fabricated via SLM and WAAM offer several notable advantages. In SLM, high dimensional accuracy (0.04–0.2 mm) and low surface roughness (2–20 μm) can be achieved. However, manufacturing efficiency is relatively constrained (180 cm^3^/h), as it is limited by scanning speed and laser power, which, when not properly optimized, can cause insufficient fusion, evaporation and pore formation [46]. In contrast, WAAM offers a significantly higher deposition rate (1000 cm^3^/h), although it is associated with comparatively lower dimensional accuracy (1–5 mm) and higher surface roughness (40–200 μm).

### 3.1. Selective Laser Melting (SLM) of Mg Alloys

Selective laser melting is a widely used method for the 3D printing magnesium alloys, owing to its utilization of a focused heat flux that enables precise energy delivery to the powder bed [24,31]. Key processing parameters, including scanning speed, laser power and layer thickness, must be carefully optimized in order to obtain dense, defect-free structures. The most commonly used feedstock materials in SLM of Mg include pure Mg, AZ-series alloys, and Mg alloys containing rare earth elements in powder form. The primary challenge associated with SLM of Mg alloys is achieving adequate densification, which directly governs the mechanical integrity of the fabricated components, as parts manufactured by SLM are inherently prone to pore formation. Table 5 summarizes the main parameters of SLM process.

**Figure 1:**
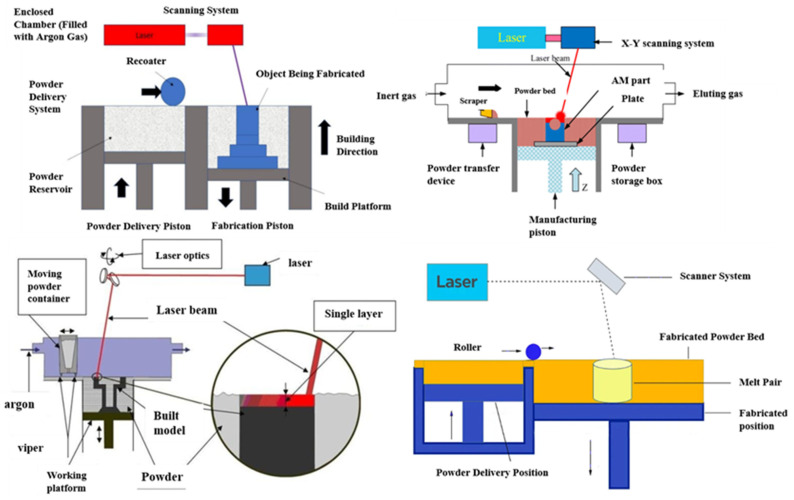
Schematic representation of SLM system.

The laser beam melts and fuses the powders to form a solid part, followed by irradiation with a laser beam to cause melting and solidification [125,126,127]. The key steps describing the SLM process begin with the design phase, where the part is designed using Computer-Aided Design (CAD) software. The most commonly used software programs are SolidWORKS, CATIA, Autodesk Fusion and Creo, which focus on creating complex geometries. The model is then converted into a STL file. In the pre-processing phase, the 3D model is sliced into thin 2D layers, and support structures are added to manage thermal stresses and overhanging features. After this, the SLM machine is prepared by filling the powder bed with metal powder, and an inert atmosphere is created in order to prevent oxidation and ignition. A thin layer of powder is then spread over the build platform. Then, a laser selectively melts the powder according to the sliced design, and the build platform is lowered by one layer thickness. The melted material solidifies rapidly, and due to the fact that the chamber is preheated, residual stress and excessive distortion is prevented. The final step involves post-processing operations, including heat treatments, Hot Isostatic Pressing (HIP), aimed at minimizing porosity and enhancing surface finishing [128,129]. Figure 1 shows the schematic representation of the SLM system.

The relative density of an SLMed part is an important factor in determining its quality. This can be defined as the ratio between the density of the SLMed material and the theoretical density of the bulk material [130]. Laser energy density is a key factor in producing optimal Mg alloy medical devices and in determining the relative density of the part. Mg alloy relative density typically ranges from 73% to 99%. The relative density of the alloys increases with increasing energy density across a wide range of alloys, including AZ91, ZK series, Mg-Gd-Zn-Zr, and Mg-9Al. For example, increasing the laser energy density from 7.5 to 15 J/mm^3^ raised the density from 74.5% to 82%. Table 6 highlights the relative density for several SLMed Mg alloys together with the corresponding laser energy density.

Porosity forms at low energy densities due to incomplete melting, which leads to large balling particles and a discontinuous trajectory. Therefore, using a higher laser energy density results in better melting of the powder, allowing it to sink between the voids of the particles and produce a smooth surface.

Hatch spacing and scanning speed are other important parameters that determine the densification of a part. Hatch spacing is the distance from the center of one beam to the center of the next beam [134]. This parameter effects not only the surface morphology but also the porosity. Studies done on AZ61 Mg alloy show that decreasing the hatch spacing from 0.1 mm to 0.06 mm, using a constant speed of 400 mm/s and a laser power of 150 W, led to a relative density was 99.4%. In contrast, at a speed of 350 mm/s, the result was unsatisfactory: the relative density was lower due to the formation of thermal stress in the samples, which led to microcracks. In conclusion, it was determined that the hatch spacing and scanning speed play a crucial role in pore size and pore shape [135,136]. Layer thickness is another parameter that optimizes the porosity and reinforces layer bonding and densification; it also affects dimensional accuracy, tensile strength and elongation [137]. It was observed [137] that preheating and using a layer of 0.15–0.25 mm, a flatter surface with no holes or pores was achieved. In contrast, using a layer thickness of 0.3 mm, with and without preheating, caused disrupted and irregular specimens. At a low layer thickness, when the same amount of energy was applied, the result showed less dense parts with voids and pores, since the laser energy density was insufficient to completely melt the powders.

Mg has low oxidation resistance at a temperature above 400 °C, which remains a challenge and restricts its applications. Magnesium alloys in the form of powders, dust, or fine chips are highly susceptible to ignition upon contact with an electric spark or open flame. To improve ignition resistance and oxidation stability, alloying Mg with calcium (Ca) and rare earth elements including Y, Gd, Ce, and Nd represents an effective strategy [138,139]. Mg-RE alloys enhance oxidation resistance through improvements in the plasticity of the oxide film and changes in microstructure. Calcium is able to suppress the continuous oxidation of Mg alloys at high temperatures. This effect is explained by microstructural changes that transform the oxide layer from porous to compact. For the formation of an effective protective oxide film, a Ca content exceeding 1 wt.% is required in Mg alloys; however, increasing the Ca content beyond this threshold is associated with a deterioration in mechanical properties. When Y is added to Mg-Ca, a flexible protective film forms, and with a content of Y above 10 wt%, the powder did not burn even at temperatures exceeding 900 °C [140]. Beryllium can improve the oxidation resistance of Mg because it acts as a surface-active element in molten magnesium. The improved oxidation resistance can be explained by the fact that the outer layer contains MgO, while the inner layer contains a mixture of MgO and BeO, which acts as a diffusion barrier.

Densification of SLMed Mg parts is the process of obtaining a dense structure with excellent properties, free of defects such as balling or loss of structural integrity, through fully melted and solidified powders. Densification increases the bonding strength between layers and minimizes gas impurities. The literature reports that achieving dense parts during the SLM process requires a higher laser energy density. For Mg-9Al, researchers concluded that higher scanning speed combined with lower laser energy density led to incomplete melting of the material, pores and balling, whereas increasing the laser energy density reduced pores and produced a smoother surface [141,142,143]. When selecting printing parameters, it is crucial to consider the requirements and intended purpose of the application and to select appropriate alloying elements in order to achieve dense parts. SLMed alloys are characterized by a uniform microstructure and refined grain structure compared with those produced by traditional methods. These advantages, which provide components with excellent mechanical properties, result from controllable process parameters, such as cooling/heating rate, orientation and the ability to manipulate input features, including rapid cooling rate, heating rate, directional solidification, and temperature. An increased scanning speed combined with decreased laser power results in lower surface energy, leading to a higher cooling rate and, ultimately, a finer microstructure in SLMed Mg alloys. Conversely, high laser power and low scanning speed result in coarser grains and a lower cooling rate.

Mechanical properties, such as hardness, tensile strength, Young’s module, elongation, fatigue and fracture behavior, are all parameters that determine the appropriate application for a material. Magnesium is a biocompatible material with an elastic modulus (3–20 GPa) similar to that of human bone. This feature is also valid for SLMed Mg alloys, which have an elastic modulus of (20.8–38.2 GPa), values that are close to that of human bone. Powder thickness and properties, as well as laser energy density, all directly affect the elastic modulus of SLMed Mg alloys. Via SLM, the average grains size is 1–15 μm, which is considerably lower than in traditional processing methods, such as casting. Microhardness in different regions is directly affected by microstructure refinement; as a result, the central region of the molten pool has higher microhardness than the edge area [144]. Studies show that direction/orientation has a major impact on mechanical properties, particularly tensile strength, highlighting the anisotropy of Mg alloys during the SLM process. Samples fabricated parallel to the building direction showed higher values than those fabricated perpendicular to it.

Post-processing and treatment of AM Mg implants and scaffold represent an important step toward enhancing overall mechanical performance and achieving the optimal requirements for their intended application. Considerable internal stress accumulates in the structure due to the layer-by-layer process and the high cooling rate of AM. Post-processing is used to reduce thermal stress and to refine the microstructure. Post-treatments fall into two categories: heat treatment and surface treatment [145]. Heat treatment, on the one hand, enhances the performance of a printed device by modifying the microstructure, and on the other hand, reduces microstructural defects and thermal stress. In SLM, the temperature distribution varies significantly from the inner area to the outer regions, resulting in different temperature gradients and, ultimately, anisotropic microstructures. Porous magnesium scaffolds are prone to early loss of mechanical integrity; this drawback was studied by Kopp et al. [146], who applied two post-processing methods to an SLM-printed Mg scaffold, plasma electrolytic oxidation (PEO) and Hot Isostatic Pressing (HIP). PEO treatment helped to reduce the degradation rate and enhance mechanical performance, whereas HIP had a detrimental effect. Although HIP initially helped densify the high porosity regions, its effect on small pores was insignificant. Obtaining a smooth surface enhances the corrosion resistance and prevents fatigue failure [147]. Roughness is a parameter that directly influences the interaction between scaffold and cell; thus, surface treatment can enhance the overall performance of the printed device. There is a wide range of surface treatment methods, such as plasma spray, physical/chemical vapor deposition, ion implantation, electrochemical oxidation, sol–gel etc. For scaffolds, particularly due to their porous structure, two approaches can deliver optimal results: chemical etching and anodization treatments, which are chemical processes, and coating the scaffold surface with bioactive ceramics [145].

### 3.2. Wire Arc Additive Manufacturing (WAAM) of Mg Alloys

Compared to other 3D printing methods, wire arc additive manufacturing (WAAM) uses metal wires and has higher deposition rates and lower manufacturing costs. This method is also able to print large-size components, and, in terms of process stability, is more reliable [148,149,150]. Table 7 summarizes the main characteristic parameters of the WAAM process.

**Figure 2:**
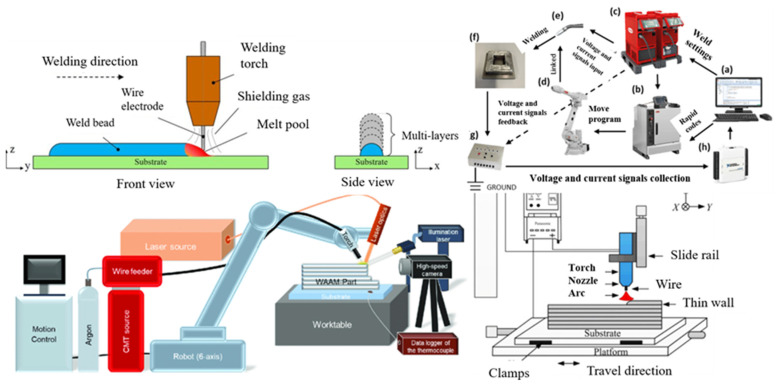
Schematic representation of the WAAM system.

Printing Al alloys via WAAM has been well researched, and its feasibility has been proven. However, there is a lack of research and studies on Mg alloys in combination with WAAM. Cold metal transfer (CMT) is a better alternative to WAAM due to its ability to reduce heat input. At the same time, Mg alloys tend to exhibit better forming and mechanical properties through CMT-WAAM. A schematic representation of the WAAM system can be observed in Figure 2.

The WAAM process can be split into three categories based on the arc welding technology used for melting the wire feedstock: plasma arc welding (PAW), gas metal arc welding (GMAW) and gas tungsten arc welding (GTAW) [124,151]. GTAW is also known as TIG (tungsten inert gas). An electric arc is formed between a tungsten electrode and the substrate below, and an inert atmosphere protects the weld pool from oxidation and contamination. PAW operates similarly to GTAW, but a nozzle constricts the arc, and the feedstock is melted by the plasma. In GMAW, also known as MIG (metal inert gas), the electric arc is generated between a filler metal and the substrate.

WAAM presets a complex physical process, and its success depends on a number of factors such as heat transmission to the wire, welding technique, wire melting and formation of the droplets that collide with the previous substrate [152]. The most common defects observed on WAAM printed parts are usually, cracks, oxidation, roughness, delamination and porosity. Therefore, in order to obtain high-quality parts, current, voltage, torch travel speed, wire speed, gas composition and flow rate, heat input, and diameter of the welding electrode are parameters that need to be taken into consideration [151]. Heat input plays a major role in achieving a smooth surface finish, a fine microstructure and good mechanical properties. Heat input is defined in Equation (1), where η is the efficiency, U is voltage, I is the current and TS is the travel speed.

(1) $$HI=\frac{\eta \;UI}{TS}$$

In recent years, the use of WAAM Mg alloys has attracted interest due to their limited plasticity, a challenge that cannot be overcome by traditional deformation-based techniques, such as forging or rolling, as well as shrinkage and internal porosity caused by casting. Currently, the majority of studies on WAAM Mg alloys focus on Mg-Al-Zn and AZ series alloys [153,154]. WAAM wires are produced via continuous casting, hot extrusion or drawing. They must provide a smooth surface, free of cracks, oxide pits or twists, and their composition should be homogeneous and free of segregation. Defects in WAAM Mg parts can be divided into two groups: process-related defects, which include roughness and residual stress, and material-related defects, which include porosity and cracks.

The corrosion resistance of WAAM-processed Mg alloys is strongly dependent on the microstructure. This challenge can be addressed from two main directions: the first involves controlling the WAAM parameters and examining how they affect grain size, and the composition and distribution of precipitates within the α-Mg matrix, the second involves post-processing steps, particularly heat treatment, intended to refine the microstructure. In a study by Li et al. [155], an AZ31 alloy prepared via CMT-WAAM with unmodified parameters was compared to an as-cast part. The grain structure of the WAAM part strongly influenced its corrosion resistance, which was found to be higher than that of the cast alloy. A high density of grain boundaries promotes the formation of an oxide film, which, due to grain refinement, leads to passivation and a decrease in corrosion rate. Conversely, when an oxide film does not form, the corrosion rate increases due to the high reactivity of the refined microstructures. In addition to grain size, crystallographic texture and secondary phases are factors that strongly influence the corrosion behavior of WAAM-processed parts.

The use of post-processing methods to improve specific microstructural features can enhance the overall corrosion resistance of WAAM-processed parts. The effect of heat treatment on the corrosion resistance of PAW-WAAM Mg-Gd-Y-Zr was investigated [156] in a 3.5 wt% NaCl solution. After the WAAM process, the obtained part was annealed and artificially aged. Following the aging process, the microstructure exhibited an α-Mg matrix and a small content of β-Mg_24_(Gd,Y)_5_ precipitate. The heat treatment eliminated residual stress and also improved the corrosion resistance.

In summary, in order to obtain SLM- or WAAM-manufactured components with minimal defects, the key factor is the optimization of process parameters, a fundamental step in achieving superior performance in AM medical devices.

## 4. Main Defects in SLMed and WAAMed Mg Alloys

SLM and WAAM processing parameters are difficult to control and represent the main cause of spheroidization, porosity, poor fusion, loss of alloying elements, cracks, and oxide inclusions.

Spheroidization is characterized by the shrinkage of the molten pool into spheres and indicates inadequate heat input. This phenomenon is caused by the surface tension of the molten pool being greater than that of the powder. In order to minimize spheroidization, two methods can be applied. The first involves controlling the oxygen content, which decreases the production of oxides and the formation of oxide films, leading to improved wetting behavior [157]. The second involves increasing the quantity of metal powder to improve fluidity, allowing the molten metal to spread over the substrate surface and thereby inhibiting spheroidization [158].

Porosity is a common defect of the SLM process. This issue usually arises from rapid solidification, where overflow of the gas is slow and the solubility of the gas decreases while the temperature drops, thus inhibiting gas evacuation. Two methods can be used to inhibit the formation of porosity. First, controlling the amount of gas elements and choosing appropriate processing parameters, and second, closely analyzing the gas escape process with consideration of thermodynamics and dynamics [159].

Poor fusion appears when the cohesion of the melting powder/wire is incomplete during the liquid-state AM process. In SLM, this defect is caused by insufficient energy from the heat source: when the thickness of the overlapping area does not reach the metal powder, some powder remains incompletely melted, resulting in poor fusion. Choosing appropriate parameters to obtain suitable energy input can successfully suppress this defect. Also, the WAAM process is also susceptible to poor fusion due to insufficient energy input and warping of the wire. Using feedstock with good formability and selecting appropriate parameters are essential to achieving accuracy and avoiding poor fusion.

Cracks represent a detrimental defect in the liquid-state AM process, decreasing the overall performance of a part. Cracks are mainly caused by residual and thermal stress, microstructure stress and constraint stress [160,161]. Solidification shrinkage of the material is also a related cause of cracking. Several methods can be used to reduce the occurrence of cracks in AM parts, including using raw materials with good plasticity, decreasing the energy input and preheating the substrate to reduce temperature.

Oxide inclusions are thin films on the surface of the part during the welding process, originating from the surface of the raw materials. These defects cause spheroidization, lack of bonding between layers and microstructure disruptions, which lead to poor mechanical properties [130,162]. Although Mg itself oxidize easily, and oxidation is difficult to avoid completely, choosing appropriate manufacturing parameters, such as a higher heat input, can minimize the formation of oxide films.

All these defects have a negative impact on the overall performance of AM Mg alloys. Therefore, from the beginning of the manufacturing process, close attention to the input parameters, the quality of the feedstock, and appropriate post-processing operations is necessary to obtain devices with excellent mechanical, physical and chemical properties.

## 5. Post-Processing of AM Mg Alloys

Medical devices manufactured by AM are prone to various defects, including but not limited to pores, residual stress, microstructural defects and mechanical anisotropy. Therefore, in order to create parts that are suited to and meet the requirements of their intended application, post-processing methods such as HIP, heat treatment and mechanical processing, among others, are needed, in addition to choosing input parameters that can reduce some of these defects. Table 8 summarizes the effect of heat treatment of AM Mg alloys [125].

### 5.1. Heat Treatment (HT)

Even though in an AM Mg alloys, some defects such as porosity, poor fusion, cracks or inhomogeneity are inevitable, post-processing techniques can be used to minimize them. Heat treatment can effectively relieve residual stress, refine the microstructure and facilitate the formation of precipitates, thus significantly improving mechanical and corrosion properties. Common heat treatment used for AM Mg alloys are solution heat treatment (T4), solid solution followed by aging heat treatment (T6) and direct aging treatment (T5). During this process, alloys are heated to the single-phase region and held there until the second phase is fully dissolved. Next, rapid quenching is employed in order to obtain a saturated solid solution and refine microstructure. The final step is aging heat treatment. Once the heat treatment is complete, the results show the formation of fine precipitates of the second phase along with an improvement in the mechanical and corrosion properties. In a study performed by Guo et al. [10], the effect of HT on the microstructure and mechanical behavior of a WAAMed AZ80M part was investigated. The AZ80M Mg alloy underwent a series of HTs, including solution HT at a temperature of 410 °C for 1.5 h, direct aging at 180 °C for 25 h, and solution HT followed by aging at 410 °C for 1.5 h followed by 180 °C for 25 h. After the T4 treatment, the majority of the dendritic second phase, characterized by low thermal stability fully dissolved; however, the second phase containing Ca and Y remained undissolved due to its high thermal stability. T5 heat treatment resulted in the formation of additional precipitates within the existing second phase. For T6 treatment, the solution heat treatment led to the dissolution of the majority of the second phase, while aging contributed to the formation of fine, continuous precipitates. Moreover, T4 heat treatment showed a significant increase in elongation due to the dissolution of the secondary phases, while T5 and T6 improved the strength of the WAAM-processed AZ80M.

Heat treatment remains the most widely employed post-processing method for additively manufactured (AM) magnesium alloys because of its versatility and effectiveness in alleviating defects such as residual stresses and microstructural inhomogeneity. Nevertheless, despite its demonstrated benefits, heat treatment also presents several challenges, including process parameter optimization, dimensional distortion, surface quality degradation, material compatibility issues, treatment non-uniformity, and increased processing cost and time. Achieving optimal heat-treatment performance therefore requires careful consideration of these factors in conjunction with material composition, component geometry, and the targeted properties. This highlights the need for comprehensive process planning and precise execution throughout AM production.

### 5.2. Hot Isostatic Pressing (HIP)

Porosity is an inherent characteristic of as-built additively manufactured (AM) magnesium alloys, negatively affecting their mechanical and corrosion performance. Among the available post-processing methods, hot isostatic pressing (HIP) is one of the most widely adopted techniques for porosity reduction. During HIP, the material is subjected to elevated temperature and high isostatic pressure within an inert atmosphere, promoting pore deformation and collapse, which ultimately leads to material densification [165,166]. In addition to reducing porosity, HIP is effective in reducing residual stresses, homogenizing the microstructure, and improving ductility and strength. The technique has been extensively applied to enhance the properties of additively manufactured SS316L [167], aluminum alloys [168], titanium alloys [169], and nickel-based alloys [170]. Nevertheless, its application as a post-processing strategy for AM magnesium alloys remains relatively limited. Gangireddy et al. investigated [171] the effect of HIP treatment on WE43 alloy with different degrees of porosity. HIP was performed at 350 °C and pressure of 104 MPa for 2 h. The results showed a significant reduction in porosity from 12.4% to 2.7% after HIP treatment. Additionally, due to the combined effect of temperature and pressure, HIP also influenced the microstructure of the parts: dissolution of the Y phase, precipitation of the Zr phase and a transformation in the shape of Nd precipitates were observed.

Hot isostatic pressing (HIP) has demonstrated considerable effectiveness in promoting pore closure and material densification, thereby offering significant potential for improving the mechanical performance of additively manufactured (AM) magnesium alloys. Despite its numerous advantages, HIP is associated with several limitations: grain coarsening and the presence of collapsed pores that may act as crack initiation sites. Furthermore, HIP requires substantial energy input and specialized equipment, leading to increased operational costs and extended processing times. Achieving uniform densification in components with complex geometries or large dimensions also remains a significant challenge, which may result in inconsistent mechanical properties throughout the fabricated part.

### 5.3. Friction Stir Processing (FSP)

Post-processing techniques, such as heat treatment and hot isostatic pressing, share a common limitation, namely the formation of abnormal grain growth at high temperatures, which can lead to a reduction in the tensile strength of the material. In addition, the fine and heterogeneous grain structures characteristic of as-built alloys, formed under rapid solidification conditions, exhibit greater susceptibility to grain coarsening than conventionally cast materials subjected to comparable temperatures and holding times [172]. Consequently, the development of innovative post-processing methods is essential to minimize defects and homogenize the microstructure while improving ductility without compromising material strength.

Friction stir processing (FSP), a severe plastic deformation technique derived from friction stir welding (FSW), has emerged as a promising alternative. FSP utilizes elevated temperatures in combination with high strain and strain rates to induce intense plastic deformation, thereby promoting grain refinement, microstructural homogenization, fragmentation of secondary phases, and elimination of defects such as porosity [173]. Numerous studies have demonstrated the effectiveness of FSP in producing refined and homogeneous microstructures in as-cast magnesium alloys, leading to significant improvements in mechanical performance [174].

However, the application of FSP as a post-processing technique for additively manufactured alloys remains largely restricted to flat geometries. Previous investigations have explored the feasibility of FSP for post-processing additively manufactured titanium alloys [175] and aluminum alloys. More recently, several studies have also examined the use of FSP as a post-processing approach for AM magnesium alloys [176,177].

## 6. Applications of Mg Alloys

The outstanding qualities of magnesium alloys, especially their light weight, mechanical properties and controllable biodegradability, make them a great choice for a number medical fields. Together with 3D printing methods, custom devices with specific properties and requirements can be achieved in order to meet the individual needs of every patient.

### 6.1. Orthopedic Implants

Traditional orthopedic implants are commonly made from stainless steel, Ti and Co-Cr alloys. Due to their non-degradability, they can cause stress shielding, and a second surgery is often needed for removal [178]. Mg alloys, particularly in combination with La or Ce, can provide excellent results for orthopedic implants, where the degradation rate matches the time, it takes for bones to fully heal. This reinforces the capability of Mg alloys to offer sufficient mechanical support during the healing process. Furthermore, their excellent biocompatibility can facilitate a more successful recovery [179]. Pure Mg, Mg-Y-RE-Zr and Mg-Ca-Zn alloys have achieved regulatory approval in several countries, such as China, Germany and South Korea [180].

Mg-based metals in orthopedy can be divided in two categories. The first is bone fixation devices, which are used in the early stages of fracture healing to provide support as the fracture heals and remodels, while the implant gradually degrades [181]. The second category is bone tissue engineering scaffolds, which are used in combination with cells into the affected area. During the degradation process, these scaffolds guide cell proliferation and differentiation, thereby stimulating bone formation and the regeneration of bone tissue [106,182].

### 6.2. Stomatology

Traditional dental implants often cause biocompatibility problems and exhibit long-term resistance in the oral environment.

Alveolar bone defects can lead to tooth extraction or implant failure. Therefore, in order to minimize these issues, Guided Tissue Regeneration (GTR) was introduced [183]. This method focuses on placing a physical barrier to separate periodontal tissues, preventing the ingrowth of connective tissue, while promoting tissue regeneration with the help of guiding cells.

For example, Mg-RE alloys can provide excellent biocompatibility and corrosion resistance, meaning that the risk of inflammation or rejection is significantly lower. Another important aspect is the ability of the scaffold used for bone regeneration to be tailored to the patient’s unique healing process [184]; therefore, the dental recovery process will be more efficient and less intrusive [179].

### 6.3. Cardiovascular Applications

Currently, most stents are composed of permanent materials, such as Co-Cr or stainless steel. These materials have to remain in the body for a long period of time and can occasionally cause difficulties. When designing a vascular stent, requirements including mechanical performance, controlled degradation and vascular biocompatibility should be primarily taken into consideration. From a mechanical standpoint, stents should resist recoil and shrinkage while maintaining structural integrity for 3–6 months in order to provide vessel patency [180]. In terms of biodegradation, an optimal degradation rate for a stent should not exceed 0.02 mm∙y^−1^, with complete degradation occurring within 12–24 months to allow for vessel remodeling. Moreover, the implant should not cause toxicity or an inflammatory response [185]. In a study performed by Chen et al. [186] an Mg-Nd-Zn-Zr alloy stent was investigated. The material was optimized using a surface coating and then implanted in a New Zealand white rabbit. CT and optical coherence tomography (OCT) images revealed no signs of thrombosis or restenosis at 5 months post-implantation, thus demonstrating that the stent successfully supported the vessel prior to biodegradation and also enabled arterial healing.

## 7. Challenges and Future Research Directions

Regenerative medicine has gained a lot of interest in recent year, and continuous development has provided a better understanding of the design process and material requirements for various applications. Even though 3D printing methods are able to create complex shapes for many applications, many challenges have arisen. Various fields of tissue engineering require scaffolds with varying properties and structures. These scaffolds should mimic natural tissue while providing good biocompatibility and the capacity to support new bone formation and the recovery of new cells. 3D printing is able to save a large number of resources, but industrial production remains a challenge. Another aspect is that creating personalized medical devices takes a lot of time; however, automating parameter selection via artificial intelligence can be a solution [187,188,189,190]. Future research should focus on increasing the functionality and diversity of applications for scaffolds as technology progresses [191,192,193].

Mg-based alloy powders exhibit high flammability, chemical reactivity, and a tendency to evaporate due to the low boiling point of Mg [194,195]. Other challenges regarding 3D printing of Mg alloys include low vaporization temperatures, high vapor pressure, and high tendency toward oxidation. However, powders of WE43, AZ91D alloys and pure Mg are commonly available [196].

Health and safety are major concerns with Mg alloys. Therefore, ensuring safety in the mass production of AM Mg alloy applications is a key priority. These measures include production in a well-controlled environment, use of minimal powder volumes, safe storage of the feedstock away from any ignition source, and a permanently controlled and monitored environment. In general, resolving these relevant challenges in the development of AM Mg alloy parts will contribute to creating higher-performance and more accurate biodegradable Mg-based alloys for the biomedical field.

To improve the additive manufacturing of biomedical magnesium alloys, several important challenges need to be addressed. Process stability remains essential due to magnesium’s high reactivity, flammability, and low vaporization temperature, which require stricter parameter control. Future research should focus on developing real-time process monitoring systems and leveraging machine learning to optimize parameters, reducing reliance on trial-and-error methods. Designing AM-specific magnesium alloy compositions is equally vital, as most current alloys were developed for conventional manufacturing and are not suited for the rapid solidification inherent to the SLM and WAAM processes.

Corrosion behavior under physiological conditions, especially in complex porous structures, is still not well understood. Promising areas for research include multifunctional surface coatings that offer controlled biodegradation along with antibacterial and osteoinductive properties, and predictive degradation models that that account for microstructural heterogeneity and residual stresses. Post-processing methods also require improvement; current techniques such as heat treatment and HIP produce uneven results in complex geometries, and applying friction stir processing to non-planar AM parts deserves further exploration.

From a clinical and regulatory perspective, long-term in vivo studies and controlled clinical trials are necessary to validate the safety and effectiveness of AM magnesium implants across various anatomical applications. To improve scalability and economic feasibility, safer feedstock handling, more efficient enclosed AM equipment, and streamlined post-processing workflows are needed. Progress in these interconnected areas is expected to accelerate the clinical adoption of personalized AM magnesium-based medical devices.

## 8. Conclusions

In recent decades, the demand for lightweight structures has increased substantially across biomedical, automotive, aerospace, and electronic applications. Magnesium and its alloys have emerged as promising candidates owing to their low density, favorable mechanical properties, excellent thermal conductivity, thermal stability, and good machinability. In particular, the biocompatibility and biodegradability of magnesium make it highly attractive for the development of next-generation medical devices and implants.

Additive manufacturing (AM) has transformed the design and fabrication of complex and patient-specific medical devices by enabling high geometric freedom, reduced material waste, and customizable processing conditions. Among the available AM technologies, selective laser melting (SLM) and wire arc additive manufacturing (WAAM) have shown considerable potential for processing magnesium alloys. SLM generally enables the fabrication of components with finer microstructures, higher dimensional accuracy, and superior mechanical strength due to the rapid solidification. In contrast, WAAM offers higher deposition rates, lower production costs, and greater suitability for manufacturing large-scale components, although it is often associated with coarser microstructures and lower surface quality.

Despite these advantages, the additive manufacturing of magnesium alloys remains challenging because of their high oxidation tendency, flammability, and chemical reactivity during processing. Nevertheless, recent studies have demonstrated significant improvements in the mechanical performance of AM Mg alloys through process optimization and post-processing treatments, leading to enhanced strength, ductility, and microstructural homogeneity.

This review highlights recent advances in the additive manufacturing of magnesium alloys, with particular emphasis on SLM and WAAM technologies, their processing characteristics, mechanical behavior, and potential applications. Future research should focus on improving process stability, minimizing defects such as porosity and cracking, developing new magnesium alloy compositions tailored for AM, and optimizing post-processing strategies to further enhance the mechanical and corrosion performance of fabricated components.

## Figures and Tables

**Table 1:** Mechanical properties of different Mg alloys fabricated via SLM and WAAM—a summary.

Alloy	Hardness (HV)	YS (MPa)	UTS (MPa)	Elongation (%)	Ref.
Selective Laser Melting (SLM)					
Pure Mg	60–89				[43]
AZ31B	64	183	212	7.9	[44]
AZ61		216	239	2.1	[45]
		233	287	3.1	
AZ91D	85	237	254	2	[46]
	100	274	296	1.8	
AZ91D		265	298	2.0	[47]
AZ91D		242	361	8.9	[48]
AZX912		253	332	3.2	[49]
Mg-9Al	66–85				[50]
WE43		194	312	14	
WE43		215	251	2.6	[51]
WE43		296	308	11.9	[52]
WE43		236	313	9.6	[53]
		275	337	10.5	
ZK60	70.1–85				[54]
GZ112K		332	351	8.6	[55]
GWZ1031K		310	347	4.1	[56]
T4		255	328	10.3	
T6		316	400	2.2	
G10K	79.8	180	228	2.2	[57]
G10K-FSP	86.2	202	272	7.5	
Wire arc additive manufacturing (WAAM)					
AZ31-GT		94	190	13	[58]
		109	222	20	
AZ31-CMT		131	210	10	[59]
		71	151	7	
AZ31-CMT		85	225	28	[60]
		125	210	17	
AZ31-GT		104	263	23	[61]
AZ31B-MIG		95	239	21	[62]
AZ31-UFP			203	20	[63]
			211	22	
ATZM31-CMT		119	211	17.9	[64]
		94	207.7	13.9	
AZ61A-CMT		99	256	15	[65]
		104	264	15	
AZ80M-GT		119	237	12	[66]
		146	308	15	
AZ80M-GT			230	13	[10]
			280	15	
AZ91-CMT			250	17	[67]
			245	16	
AZ61A			275	16.8	[68]
			272	17.2	
AEX11-CMT			243	4.5	
			233	5.2	
GW63K-UFP		151	237	8.9	[69]
		150	232	8.3	
GWZ1031K		150	247	3.3	[70]
		154	271	8.7	
T6		214	337		
		215	331		
GWZ421K		121	224	11.4	[71]
		123	224	12.7	
T4		114	229	16	
		116	234	17.7	
T6		157	285	16.2	
		157	288	17.1	

**Table 2:** Biodegradability of SLMed Mg alloys.

Alloy	Powder Shape	Particle Size (μm)	Processing	Biodegradation Rate	Ref.
AZ61	spherical	70	SLM	12.26 mg/cm^−2^	[95]
ZK60	spherical	50	SLM	0.006 mL cm^−2^ h^−1^	[96]
ZK30-xAl	spherical	ZK30—45–74; Al—5–15	SLM	0.17 ± 0.02 mg cm^−2^ day^−1^	[97]
Mg	irregular	75–150	SLM	Fail	[98]
WE43	spherical	25–60	SLM	0.17 mL/cm^2^	[99]
ZK60-Cu	spherical	ZK30: 50; Cu: 80 nm	SLM	Close to 1.01 mmy^−1^	[100]

**Table 3:** Corrosion properties of WAAMed Mg Alloys.

Alloy	Type of WAAM Process	Processing Parameters	Corrosion Properties	Ref.
AZ31	GTAW	Current—75 A; wire feeding speed—2500 mm/min; travel speed—250 mm/min; pulse frequency—200 Hz; arc length—3 mm; argon flow rate—20 L/min	Electrolyte = 3.5 wt%; NaCl solution at room temperature; E_corr_ = −1.6 V_SCE_; i_corr_ = 3.43 μA/cm^2^	[58]
AZ31	CMT-GMAW	Current—105 A; arc voltage—14.2 V; wire feeding speed—7404 mm/min; travel speed—600 mm/min; distance from substrate to torch nozzle—10–12 mm; argon flow rate—20 L/min	Electrolyte = 3.5 wt%; NaCl solution at room temperature; E_corr_ = −1.6 V_SCE_; i_corr_ = 1.373μA/cm^2^	[101]
AZ31	CMT-GMAW	Current—80 A; arc voltage—20.5 V; wire feeding speed—800 mm/min; travel speed—500 mm/min; distance from substrate to torch nozzle –12 mm; argon flow rate—18 L/min	Electrolyte = Hanks’ solution at 37 °C; E_corr_ = −1.48 V_SCE_; i_corr_ = 3.8 μA/cm^2^ (middle cross section)	[102]
Mg-10Gd-4Y-2Zn-0.5Zr (GWZ1042K)	CMT-GMAW	Current—160 A; arc voltage—13 V; wire feeding speed—1400 mm/min; travel speed—900 mm/min; argon flow rate—25 L/min	Electrolyte = 3.5 wt%; NaCl solution at room temperature; E_corr_ = −1.65 V_SCE_; i_corr_ = 76.6 μA/cm^2^, after 1 h of immersion	[103]
AZ31	CMT-GMAW	Wire feeding speed—5500–7000 mm/min; travel speed—400–600 mm/min; argon flow rate—20 L/min	Electrolyte = 3.5 wt%; NaCl solution at room temperature; E_corr_ = −1.52 V_SCE_; i_corr_ = 47.04 μA/cm^2^	[104]

**Table 4:** Advantages, disadvantages and applications of AM methods for Mg alloys—a summary.

Method	Advantages	Disadvantages	Applications
Selective Laser Melting (SLM)	-Dimensional accuracy and excellent surface finish -Superior mechanical performance -Removal of some post- processing methods -Near-full-density parts achievable under optimized process parameters (laser power, scanning speed, hatch spacing, and layer thickness) -Low surface roughness	-Increased residual stress -Expensive -Instability of the molten pool -Sensitive to corrosion	-Biomedical field (cardiovascular stent, bone implants, bone scaffolds) -Automobile industry -Aerospace industry -Defense industry
Selective Laser Sintering (SLS)	-High dimensional accuracy -Good mechanical properties -Low anisotropy -Removal of any post-curing treatment -Reduced need for support structures compared to other powder bed fusion processes, although support may still be required for complex overhanging geometries -No need for support structures	-Poor surface quality -Warping and shrinkage caused by thermal distortion -Low tensile strength -Poor surface roughness	-Biomedical field (bone implants, bone scaffolds) -Automotive industry -Aerospace industry -Military industry -Electronics industry
Directed Energy Deposition (DED)	-Increased deposition rate and material efficiency -Allows the creation of large and complex parts -Minimizes the need for post-processing operations	-Limited options for compatible Mg alloy wires/feedstock -High oxidation during DED process, which leads to a negative effect on the material properties -Porosity and microstructural defects cause strength and performance issues -Specialized equipment is needed for safe operation due to magnesium reactivity	-Aerospace industry -Defense industry -Automotive industry -Oil and gas industry
Binder Jetting	-Inexpensive method -Fast process -Wide range of materials -Able to function at low temperature and pressures -No need for post-processing	-Removing the binder completely is an intricate process -Elaborated components are brittle due to the glued particles, thus limiting the mechanical performance -Post-processing is needed -Low precision	-Biomedical fields (implants, scaffolds) -Food industry -Molds and sand casting -Construction industry
Indirect Additive Manufacturing	-Removes explosive Mg powder and is much safer -Alternative in developing Mg components by avoiding the safety risks -Excellent surface quality	-Restricted to macro-scale features -Inconsistency between design and finished part	-Clinical fields, such as scaffolds implants, nasal prothesis -Microvascular networks
Laser Powder Bed Fusion (LPBF)	-A large field of applications -Dimensional accuracy and good densification -Fine-grained microstructures, good mechanical properties due to rapid solidification	-Processing parameters can cause into chemical composition and mechanical properties of the finished part -Limited availability of Mg feedstock	-Medical and dental (artificial hips/bones, crowns) -Automotive and motorsport industry -Oil and gas industry
Wire Arc Additive Manufacturing (WAAM)	-No limitation regarding part size -Alternative in developing Mg components by avoiding the safety risks -Low cost -High deposition rates and efficiency	-Low dimensional stability in comparison to LPBF -Limited availability of custom alloys -Coarser grains -Geometrical limitation due to the surface tension	-Transportation industry -Biomedical industry
Friction Stir Additive Manufacturing (FSAM)	-Capable of creating large parts -Usage of a variety of materials -Ability to produce variable microstructures for different applications	-Scale and speed production are limited by the machine dimensions -Issue in clamping the material	-Railway industry -Aerospace industry -Medical and defense industry -Renewable industry

**Table 5:** Main parameters of SLM process.

Parameters	SLM
Power	Laser: 0.1–1 kW
Raw materials: size	Powder: 5–80 μm
Temperature gradient	10^6^–10^7^ K/m
Cooling rate	10^5^–10^7^ K/s
Built efficiency	180 cm^3^/h
Part size	600^3^ mm^3^
Part accuracy	0.04–0.2 mm
Part roughness	2–20 μm

**Table 6:** Relative density and laser energy density of SMLed Mg alloys.

Alloy	Laser Energy Density J/mm^3^	Relative Density %	Ref.
Pure Mg	300	96.13	[131]
Mg-9Al	187.5	82	[50]
Mg-1Zn	183.67	99.35	[132]
AZ61	156.25	99.4	[45]
AZ91D	166.7	99.52	[46]
WE43	55.55	99.48	[133]

**Table 7:** Main parameters of WAAM process.

Parameters	WAAM
Power	Arc: 1–5 kW
Raw materials: size	Wire: ⌀ 1–5 mm
Temperature gradient	10^3^–10^4^ K/m
Cooling rate	10^3^–10^2^ K/s
Built efficiency	1000 cm^3^/h
Part size	3000^3^ mm^3^
Part accuracy	1–5 mm
Part roughness	40–200 μm

**Table 8:** Summary of heat treatment for SLMed and WAAMed Mg alloys.

Mg Alloy	AM Method	Heat Treatment	Results	Ref.
WE43	SLM	Solutionizing at 536 °C for 24 h followed by aging at 536 °C for 48 h	After the heat treatment, the strength remained approximately the same and an improvement in tensile strain was observed.	[51]
Mg-Al-Zn with different content of Ag	SLM	1. Solutionizing at 420 °C for 6–8 h followed by aging at 200 °C for 10 h 2. Solutionizing at 420 °C for 6–8 h	Decrease in mechanical properties and improvement in corrosion resistance after solutionizing. Improvement in mechanical properties and corrosion resistance after solutionizing followed by aging.	[163]
Mg-10Gd-0.2Zr	SLM	1. Aging at 200 °C 2. FSP followed by aging at 200 °C	Excellent mechanical behavior after FSP treatment followed by aging in comparison to only aging.	[57]
AZ91	WAAM	Solution annealing at 415 °C for 8 h	Grain coarsening, removal of non-basal orientation and dissolution of Mg_17_Al_22_ and increased corrosion resistance after annealing	[164]
AZ80M	WAAM	1. Solutionizing at 410 °C for 1.5 h 2. Aging at 180 °C for 25 h 3. Solutionizing at 410 °C for 1.5 h followed by aging at 180 °C for 25 h	Improvement in strength and ductility, elimination of anisotropy after solutionizing followed by aging. The highest ductility was obtained after solutionizing followed by aging and uniform distribution of hardness in comparison to aging.	[10]

## Data Availability

No new data were created or analyzed in this study. Data sharing is not applicable to this article.

## References

[1] Mao L., Hu Z., Song C. (2025). Biodegradable Mg-based devices: From passive support to active modulation. J. Magnes. Alloys.

[2] Antoniac I., Manescu (Paltanea) V., Paltanea G., Antoniac A., Nemoianu I.V., Petrescu M.I., Dura H., Bodog A.D. (2022). Additive Manufactured Magnesium-Based Scaffolds for Tissue Engineering. Materials.

[3] He M., Chen L., Yin M., Xu S., Liang Z. (2023). Review on magnesium and magnesium-based alloys as biomaterials for bone immobilization. J. Mater. Res. Technol..

[4] Salhotra A., Shan H.N., Levi B., Longaker M.T. (2020). Mechanisms of bone development and repair. Nat. Rev. Mol. Cell Biol..

[5] Li J., Qin L., Yang K., Ma Z., Wang Y., Cheng L., Zhao D. (2020). Materials evolution of bone plates for internal fixation of bone fractures: A review. J. Mater. Sci. Technol..

[6] Claes L., Recknagel S., Ignatius A. (2012). Fracture healing under healthy and inflammatory conditions. Nat. Rev. Rheumatol..

[7] Lacin N., Sfeir C. (2026). Magnesium-Based Resorbable Biomaterials: Biological Effects to Clinical Use. J. Dent. Res..

[8] Cheng J., Deng Q., Wu W., Feng X., Pang Q. (2026). Study on the effect and mechanism of gold nanocoated magnesium bone scaffolds for bone repair. BMC Musculoskelet. Disord..

[9] Xiong W., Zhou Y., Yan Y., Li Y., Li J., Yuan Y., Shi X., Liu K. (2026). Magnesium metabolism: A potential breakthrough in osteoporosis intervention. iScience.

[10] Azadi A., Ebel T., Wolff M., O’Cearbhaill E., Celikin M. (2025). Additive manufacturing of magnesium alloys for biomedical applications: Critical review on sinterability and alloy development. J. Mater. Res. Technol..

[11] Amukarimi S., Mozafari M. (2021). Biodegradable magnesium-based biomaterials: An overview of challenges and opportunities. MedComm.

[12] Celikin M., Pekguleryuz M. (2012). The role of α-Mn precipitation on the creep mechanisms of Mg-Sr-Mn. Mater. Sci. Eng. A.

[13] Guo Y., Quan G., Celikin M., Ren L., Zhan Y., Fan L., Pan H. (2022). Effect of heat treatment on the microstructure and mechanical properties of AZ80M magnesium alloy fabricated by wire arc additive manufacturing. J. Magnes. Alloys.

[14] Baltatu M.S., Vizureanu P., Sandu A.V. (2024). Advences in New Functional Biomaterials for Medical Applications. Crystals.

[15] Akbarzadeh F.Z., Sarraf M., Ghomi E.R., Kumar V.V., Salehi M., Ramakrishna S., Bae S. (2024). A state-of-the-art review on recent advances in the fabrication and characteristics of magnesium-based alloys in biomedical applications. J. Magnes. Alloys.

[16] Chen Y., Xu Z., Smith C., Sankar J. (2014). Recent advances on the development of magnesium alloys for biodegradable implants. Acta Biomater..

[17] Akbarzadeh F.Z., Ghomi E.R., Ramakrishna S. (2022). Improving the corrosion behavior of magnesium alloys with a focus on AZ91 Mg alloy intended for biomedical application by microstructure modification and coating. J. Eng. Med..

[18] Bazhenov V., Koltygin A., Komissarov A., Li A., Bautin V., Khasenova R., Anishchenko A., Seferyan A., Komissarova J., Estrin Y. (2020). Gallium-containing magnesium alloys for potential use as temporary implants in osteosynthesis. J. Magnes. Alloys.

[19] Istrate B., Muteanu C., Antoniac I.V., Lupescu S.C. (2022). Current Research Studies of Mg–Ca–Zn Biodegradable Alloys Used as Orthopedic Implants—Review. Crystals.

[20] Zerankeshi M.M., Alizadeh R., Gerashi E., Asadollahi M., Langdon T.G. (2022). Effects of heat treatment on the corrosion behavior and mechanical properties of biodegradable Mg alloys. J. Magnes. Alloys.

[21] Baltatu M.S., Chiriac-Moruzzi C., Vizureanu P., Tóth L., Novák J. (2022). Effect of Heat Treatment on Some Titanium Alloys Used as Biomaterials. Appl. Sci..

[22] Bița A.I., Antoniac I., Miculescu M., Stan G.E., Leonat L., Antoniac A., Constantin B., Forna N. (2022). Electrochemical and In Vitro Biological Evaluation of Bia-Active Coatings Deposited by Magnetron Sputtering onto Biocompatible Mg-0.8Ca Alloy. Materials.

[23] Chen J., Tan L., Yu X., Etim I.P., Ibrahim M., Yang K. (2018). Mechanical properties of magnesium alloys for medical application: A review. J. Mech. Behav. Biomed. Mater..

[24] Liu Z., Wang L., Luo S., Feng Y., Zhao S., Fu Y. (2024). Effect of heat treatment on microstructure and mechanical properties of WE43 alloy fabricated by wire arc additive manufacturing. Mater. Commun..

[25] Girelli L., Tocci M., Montesano L., Pola A. (2017). Optimization of heat treatment parameters for additive manufacturing and gravity casting AlSi10Mg alloy. Mater. Sci. Eng..

[26] Cai X., Chen F., Dong B., Lin S. (2024). Effect of heat treatment on the microstructure and mechanical properties of AZ91D magnesium alloy fabricated via GTA wire arc additive manufacturing. J. Mater. Res. Technol..

[27] Karunakaran R., Ortgies S., Tamayol A., Bobaru F., Sealy M.S. (2020). Additive manufacturing of magnesium alloys. Bioact. Mater..

[28] Allavikutty R., Gupta P., Santra T.S., Rengaswamy J. (2021). Additive manufacturing of Mg alloys for biomedical applications: Current status and challenges. Curr. Opin. Biomed. Eng..

[29] Khiabani A.B., Yarmand B., Mozafari M. (2019). Emerging magnesium-based biomaterials for orthopedic implantation. Emerg. Mater. Res..

[30] Piazza S., Merrigan B., Dowling D.P., Celikin M. (2020). The effects of geometry and laser power on the porosity and melt pool formation in additively manufactured 316L stainless steel. Int. J. Adv. Manuf. Technol..

[31] Farooq M.Z., Wu Y., Zheng M., Lu L. (2025). Ignition-proof magnesium alloys with rare earth elements: A novel framework to predict combustion phases, surface morphologies, and hidden features using heating rates. J. Magnes. Alloys.

[32] Barbulică I., Mămăligă I. (2020). A study of the flammability of magnesium in absence of oxygen. Bull. Polytech. Inst. Iasi.

[33] Ibrahim H., Esfahani S.N., Poorganji B., Dean D., Elahinia M. (2017). Resorbable bone fixation alloys, forming, and post-fabrication treatments. Mater. Sci. Eng. C.

[34] Chuan K., Khan I., Malhotra R., Zhu D. (2021). Additive manufacturing and 3D printing of metallic biomaterials. Eng. Regen..

[35] Benerjee P.C., Al-Saadi S., Choudhary L., Harandi S.E., Singh R. (2019). Magnesium Implants: Prospects and Challenges. Materials.

[36] Walter R., Kannan M.B. (2011). In-vitro degradation behavior of WE54 magnesium alloy in simulated body fluid. Mater. Lett..

[37] Wang T.-S., Hua Z.-M., Yang Y., Jia H.-L., Wang C., Zha M., Gao Y., Wang H.-Y. (2024). Macro-/micro-structures and mechanical properties of magnesium alloys based on additive manufacturing: A review. J. Mater. Sci..

[38] Istrate B., Rau J.V., Munteanu C., Antoniac I.V., Saceleanu V. (2020). Properties and in vitro assessment of ZrO_2_-based coatings obtained by atmospheric plasma jet spraying on biodegradable Mg-Ca and Mg-Ca-Zr alloys. Ceram. Int..

[39] Kumar A., Choudhari A., Gupta A.K., Kumar A. (2024). Rare Earth based magnesium alloys as a potential biomaterial for the future. J. Mater. Alloys.

[40] Zhongjun W., Weiping J., Jianzhong C. (2007). Study on the Deformation Behavior of Mg-3.6%Er Magnesium Alloy. J. Rare Earths.

[41] Wu B.L., Wan G., Du X.H., Zhang Y.D., Wagner F., Esling C. (2012). The quasi-static mechanical properties of extruded binary Mg-Er alloys. Mater. Sci. Eng. A.

[42] Yan J., Sun Y., Xue S., Tao W. (2008). Microstructure and mechanical properties in cast magnesium-neodymium binary alloys. Mater. Sci. Eng. A.

[43] Seitz J.-M., Eifler R., Stahl J., Kietzmann M., Bach F.-W. (2012). Characterization of MgNd2 alloy for potential applications in bioresorbable implantable devices. Acta Biomater..

[44] Li Z., Gu X., Lou S., Zheng Y. (2008). The development of binary Mg-Ca alloys for use as biodegradable materials within bone. Biomaterials.

[45] Bakhsheshi-Rad H.R., Idris M.H., Abdul-Kadir M.R., Ourdjini A., Medraj M., Daroonparvar M., Hamzah E. (2014). Mechanical and bio-corrosion properties of quaternary Mg-Ca-Mn-Zn alloys compared with binary Mg-Ca alloys. Mater. Des..

[46] Ng C.C., Savalani M.M., Lau M.L., Man H.C. (2011). Microstructure and mechanical properties of selective laser melted magnesium. Appl. Surf. Sci..

[47] Pawlak A., Szymczyk P.E., Kurzynowski T., Chlebus E. (2020). Selective laser melting of magnesium AZ31B alloy powder. Rapid Prototyp. J..

[48] Liu S., Yang W., Shi X., Li B., Duan S., Guo H., Guo J. (2019). Influence of laser process parameters on the densification, microstructure and, mechanical properties of selective laser melted AZ61 magnesium alloy. J. Alloys Compd..

[49] Wei K., Gao M., Wang Z., Zeng X. (2014). Effect of energy input on formability, microstructure and mechanical properties of selective laser melted AZ91D magnesium alloy. Mater. Sci. Eng. A.

[50] Niu X., Shen H., Fu J., Feng J. (2021). Effective control of microstructure evolution in AZ91D magnesium alloy by SiC nanoparticles in laser powder-bed fusion. Mater. Des..

[51] Li X., Fang X., Jiang X., Duan Y., Li Y., Zhang H., Li X., Huang K. (2023). Additively manufactured high-performance AZ91D magnesium alloys with excellent strength and ductility via nanoparticles reinforcement. Addit. Manuf..

[52] Proaño B., Miyahara H., Matsumoto T., Hamada S., Sakai H., Ogawa K., Suyalatu, Noguchi H. (2019). Weakest region analysis of non-combustible Mg products fabricated by selective laser melting. Theor. Appl. Fract. Mech..

[53] Zhang B., Liao H., Coddet C. (2012). Effects of processing parameters on properties of selective laser melting Mg–9%Al powder mixture. Mater. Des..

[54] Hyer H., Zhou L., Benson G., McWilliams B., Cho K., Sohn Y. (2020). Additive manufacturing of dense WE43 Mg alloy by laser powder bed fusion. Addit. Manuf..

[55] Zumdick N.A., Jauer L., Kersting L.C., Kutz T.N., Schleifenbaum J.H., Zander D. (2019). Additive manufactured WE43 magnesium: A comparative study of the microstructure and mechanical properties with those of powder extruded and as-cast WE43. Mater. Charact..

[56] Li K., Chen W., Yin B., Ji C., Bai S., Liao R., Yang T., Wen P., Jiang B., Pan F. (2023). A comparative study on WE43 magnesium alloy fabricated by laser powder bed fusion coupled with deep cryogenic treatment: Evolution in microstructure and mechanical properties. Addit. Manuf..

[57] Kurzynowski T., Pawlak A., Smolina I. (2020). The potential of SLM technology for processing magnesium alloys in aerospace industry. Arch. Civ. Mech. Eng..

[58] Deng Q., Wu Y., Zhu W., Chen K., Liu D., Peng L., Ding W. (2022). Effect of heat treatment on microstructure evolution and mechanical properties of selective laser melted Mg-11Gd-2Zn-0.4Zr alloy. Mater. Sci. Eng. A.

[59] Deng Q., Wu Y., Wu Q., Xue Y., Zhang Y., Peng L., Ding W. (2022). Microstructure evolution and mechanical properties of a high-strength Mg-10Gd-3Y–1Zn-0.4Zr alloy fabricated by laser powder bed fusion. Addit. Manuf..

[60] Deng Q., Wu Y., Su N., Chang Z., Chen J., Peng L., Ding W. (2021). Influence of friction stir processing and aging heat treatment on microstructure and mechanical properties of selective laser melted Mg-Gd-Zr alloy. Addit. Manuf..

[61] Fang X., Yang J., Wang S., Wang C., Huang K., Li H., Lu B. (2022). Additive manufacturing of high performance AZ31 magnesium alloy with full equiaxed grains: Microstructure, mechanical property, and electromechanical corrosion performance. J. Mater. Process. Technol..

[62] Yang X., Liu J., Wang Z., Lin X., Liu F., Huang W., Liang E. (2020). Microstructure and mechanical properties of wire and arc additive manufactured AZ31 magnesium alloy using cold metal transfer process. Mater. Sci. Eng. A.

[63] Wang P., Zhang H., Zhu H., Li Q., Feng M. (2021). Wire-arc additive manufacturing of AZ31 magnesium alloy fabricated by cold metal transfer heat source: Processing, microstructure, and mechanical behavior. J. Mater. Process. Technol..

[64] Guo J., Zhou Y., Liu C., Wu Q., Chen X., Lu J. (2016). Wire Arc Additive Manufacturing of AZ31 Magnesium Alloy: Grain Refinement by Adjusting Pulse Frequency. Materials.

[65] Takagi H., Sasahara H., Abe T., Sannomiya H., Nishiyama S., Ohta S., Nakamura K. (2018). Material-property evaluation of magnesium alloys fabricated using wire-and-arc-based additive manufacturing. Addit. Manuf..

[66] Cao Q., Qi B., Zeng C., Zhang R., He B., Qi Z., Wang F., Wang H., Cong B. (2022). Achieving equiaxed microstructure and isotropic mechanical properties of additively manufactured AZ31 magnesium alloy via ultrasonic frequency pulsed arc. J. Alloys Compd..

[67] Yang Y.H., Guan Z.P., Ma P.K., Ren M.W., Jia H.L., Zhao P., Zha M., Wang H.Y. (2024). Wire arc additive manufacturing of novel ATZM31 Mg alloy: Microstructure evolution and mechanical properties. J. Magnes. Alloys.

[68] Kleim T., Arnoldt A., Schnall M., Gneiger S. (2021). Microstructure Formation and Mechanical Properties of a Wire-Arc Additive Manufactured Magnesium Alloy. J. Miner. Met. Mater. Soc..

[69] Guo Y., Quan G., Jiang Y., Ren L., Fan L., Pan H. (2021). Formability, microstructure evolution and mechanical properties of wire arc additively manufactured AZ80M magnesium alloy using gas tungsten arc welding. J. Magnes. Alloys.

[70] Bi J., Shen J., Hu S., Zhen Y., Yin F., Bu X. (2020). Microstructure and mechanical properties of AZ91 Mg alloy fabricated by cold metal transfer additive manufacturing. Mater. Lett..

[71] Gneiger S., Österreicher J.A., Arnoldt A.R., Birgmann A., Fehlbier M. (2020). Development of a High Strength Magnesium Alloy for Wire Arc Additive Manufacturing. Metals.

[72] Cao Q., Zeng C., Qi B., Jiang Z., Zhang R., Wang F., Cong B. (2023). Excellent isotropic mechanical properties of directed energy deposited Mg-Gd-Y-Zr alloys via establishing homogeneous equiaxed grains embedded with dispersed nano-precipitation. Addit. Manuf..

[73] Cao Q., Zeng C., Cai X., Zhang R., Wang F., Wang H., Zhang Y., Qi B., Cong B. (2023). High-strength Mg-10Gd-3Y-1Zn-0.5Zr alloy fabricated by wire-arc directed energy deposition: Phase transformation behavior and mechanical properties. Addit. Manuf..

[74] Li X., Fang X., Fang D., Fu W., Zhang X., Huang K. (2023). On the excellent strength-ductility synergy of wire-arc directed energy deposited Mg-Gd-Y-Zn-Zr alloy via manipulating precipitates. Addit. Manuf..

[75] Zeng Z., Salehi M., Kopp A., Xu S., Esmaily M., Birbilis N. (2022). Recent progress and perspectives in additive manufacturing of magnesium alloys. J. Magnes. Alloys.

[76] Liu D., Yang D., Li X., Hu S. (2019). Mechanical properties, corrosion resistance and biocompatibilities of degradable Mg-RE alloys: A review. J. Mater. Res. Technol..

[77] Sanchez A.H.M., Luthringer B.J.C., Feyerabend F., Willumeit R. (2015). Mg and Mg alloys: How comparable are in vitro and in vivo corrosion rates? A review. Acta Biomater..

[78] Ezhilselvi V., Nithin J., Balaraju J.N., Subramanian S. (2016). The influence of current density on the morphology and corrosion properties of MAO coatings on AZ31B magnesium alloy. Surf. Coat. Technol..

[79] Ghali E. (2010). Corrosion Resistance of Aluminum and Magnesium Alloys: Understanding, Performance, and Testing.

[80] Yao W., Wu L., Huang G., Jiang B., Atrens A., Pan F. (2020). Superhydrophobic coatings for corrosion protection of magnesium alloys. J. Mater. Sci. Technol..

[81] Chen X., Zhang J., Wang M., Wang W., Zhao D., Huang H., Zhao Q., Xu X., Zhang H., Huang G. (2024). Research progress of heterogeneous structure magnesium alloys: A review. J. Magnes. Alloys.

[82] Wu T., Zhang K. (2023). Corrosion and Protection of Magnesium Alloys: Recent Advances and Future Perspectives. Coatings.

[83] Xu S.W., Matsumoto N., Kamado S., Honma T., Kojima Y. (2009). Effect of Mg17Al12 precipitates on the microstructural changes and mechanical properties of hot compressed AZ91 magnesium alloy. Mater. Sci. Eng. A.

[84] Celotto S. (2000). TEM study of continuous precipitation in Mg–9 wt%Al–1 wt%Zn alloy. Acta Mater..

[85] Cáceres C.H., Rovera D.M. (2001). Solid solution strengthening in concentrated Mg–Al alloys. J. Light Met..

[86] Liu B.C., Zhang S., Xiong H.W., Dai W.H., Ma Y.L. (2022). Effect of Al Content on the Corrosion Behavior of Extruded Dilute Mg–Al–Ca–Mn Alloy. Acta Metall. Sin. (Engl. Lett.).

[87] Yin Z., Chen Y., Yan H., Zhou G., Wu X., Hu Z. (2019). Effects of the second phases on corrosion resistance of AZ91-xGd alloys treated with ultrasonic vibration. J. Alloys Compd..

[88] Kondoh K., Takei R., Kariya S., Umeda J. (2022). Quantitative analysis on surface potentials of impurities and intermetallic compounds dispersed in Mg alloys using scanning Kelvin probe force microscopy and ultraviolet photoelectron spectroscopy. Mater. Chem. Phys..

[89] Gu D., Wang J., Chen Y., Peng J. (2020). Effect of Mn addition and refining process on Fe reduction of Mg−Mn alloys made from magnesium scrap. Trans. Nonferrous Met. Soc. China.

[90] Metalnikov P., Ben-Hamu G., Templeman Y., Shin K.S., Meshi L. (2018). The relation between Mn additions, microstructure and corrosion behavior of new wrought Mg-5Al alloys. Mater. Charact..

[91] Kim J.I., Nguyen H.N., You B.S., Kim Y.M. (2019). Effect of Y addition on removal of Fe impurity from magnesium alloys. Scr. Mater..

[92] Cai H., Guo F., Su J., Liu L. (2019). Existing forms of Gd in AZ91 magnesium alloy and its effects on mechanical properties. Mater. Res. Express.

[93] Zhang Y., Huang Y., Feyerabend F., Blawert C., Gan W., Maawad E., You S., Gavras S., Scharnagl N., Bode J. (2021). Influence of the amount of intermetallics on the degradation of Mg-Nd alloys under physiological conditions. Acta Biomater..

[94] Cheng X., Qu Y., Kang C., Kang M., Dong R., Zhao J. (2021). Development of new Mg-Zn-Ca-Y alloy and in-vitro and in-vivo evaluations of its biological characteristics. Mater. Commun..

[95] Daloz D., Steinmetz P., Michot G. (1997). Corrosion Behavior of Rapidly Solidified Magnesium-Aluminum-Zinc Alloys. Corrosion.

[96] Izumi S., Yamasaki M., Kawamura Y. (2009). Relation between corrosion behavior and microstructure of Mg–Zn–Y alloys prepared by rapid solidification at various cooling rates. Corros. Sci..

[97] Liu S., Guo H. (2020). A review of SLMed Magnesium Alloys: Processing, Properties, Alloying Elements and Postprocessing. Metals.

[98] He C., Bin S., Wu P., Gao C., Feng P., Yang Y., Liu L., Zhou Y., Zhao M., Shuai C. (2017). Microstructure Evolution and Biodegradation Behavior of Laser Rapid Solidified Mg–Al–Zn Alloy. Metals.

[99] Shuai C., Yang Y., Wu P., Lin P., Liu Y., Zhou Y., Feng P., Liu X., Peng S. (2017). Laser rapid solidification improves corrosion behavior of Mg-Zn-Zr alloy. J. Alloys Compd..

[100] Shuai C., He C., Feng P., Guo W., Gao C., Wu P., Yang Y., Bin S. (2017). Biodegradation mechanisms of selective laser-melted Mg–xAl–Zn alloy: Grain size and intermetallic phase. Virtual Phys. Prototyp..

[101] Ng C.C., Savalani M.M., Man H.C., Gibson I. (2010). Layer manufacturing of magnesium and its alloy structures for future applications. Virtual Phys. Prototyp..

[102] Li Y., Zhou J., Pavanram P., Leeflang M.A., Fockaert L.I., Pouran B., Tümer N., Schröder K.-U., Mol J.M.C., Weinans H. (2018). Additively manufactured biodegradable porous magnesium. Acta Biomater..

[103] Shuai C., Liu L., Zhhao M., Feng P., Yang Y., Guo W., Gao C., Yuan F. (2018). Microstructure, biodegradation, antibacterial and mechanical properties of ZK60-Cu alloys prepared by selective laser melting technique. J. Mater. Sci. Technol..

[104] Goka S., Manjaiah M., Davidson M.J. (2024). Study on Mechanical, Microstructural and Corrosion Analysis of Wire Arc Additive Manufactured AZ31 Magnesium Alloy. Met. Mater. Int..

[105] Manjhi S.K., Seker P., Bontha S., Balan A.S.S. (2023). Effect of equiaxed grains and secondary phase particles on mechanical properties and corrosion behaviour of CMT-based wire arc additive manufactured AZ31 Mg alloy. CIRP J. Manuf. Sci. Technol..

[106] Zhang Z., Ji L., Wang S., Zhao Z., Wang L., Ma K., Li Y., Zhang D., Bai P. (2024). Revealing corrosion behavior and mechanism of cold metal transfer-wire arc additive manufactured Mg-10Gd-4Y-2Zn-0.5Zr alloy in 3.5 wt% NaCl. Corros. Sci..

[107] Fang X., Yang J., Jiang X., Li X., Chen R., Huang K. (2024). Wire-arc directed energy deposited high-performance AZ31 magnesium alloy via a novel interlayer hammering treatment. Mater. Sci. Eng. A.

[108] Bairagi D., Mandal S. (2022). A comprehensive review on biocompatible Mg-based alloys as temporary orthopedic implants: Current status, challenges, and future prospects. J. Magnes. Alloys.

[109] Yang Y., He C., E D., Yang W., Qi F., Xie D., Shen L., Peng S., Shuai C. (2020). Mg bone implant: Features, developments and perspectives. Mater. Des..

[110] Agarwal S., Curtin J., Duffy B., Jaiswal S. (2016). Biodegradable magnesium alloys for orthopedic applications: A review on corrosion, biocompatibility and surface modifications. Mater. Sci. Eng. C.

[111] Staiger M.P., Pietak A.M., Huadmai J., Dias G. (2006). Magnesium and its alloys as orthopedic biomaterials: A review. Biomaterials.

[112] Varmann J. (2003). Magnesium: Nutrition and metabolism. Mol. Asp. Med..

[113] Touyz R.M. (2004). Magnesium in clinical medicine. Front. Biosci. A J. Virtual Libr..

[114] Zhao D., Witte F., Lu F., Wang J., Li J., Qin L. (2017). Current status on clinical applications of magnesium-based orthopedic implants: A review from clinical translational perspective. Biomaterials.

[115] Saris N.E., Mervaala E., Karppanen H., Khawaja J., Lewenstam A. (2000). Magnesium: An update on physiological, clinical and analytical aspects. Clin. Chim. Acta.

[116] Sudha P., Tun K.S., Pillai J., Dutta M., Gupta M., Kumar V.S. (2024). Biocorrosion and Cytotoxicity Studies on Biodegradable Mg-Based Multicomponent Alloys. Bioengineering.

[117] Liu J., Bian D., Zheng Y., Chu X., Lin Y., Wang M., Lin Z., Li M., Zhang Y., Guan S. (2020). Comparative in vitro study on binary Mg-RE (Sc, Y, La, Ce, Pr, Nd, Sm, Eu, Gd, Tb, Dy, Ho, Er, Tm, Yb and Lu) alloy systems. Acta Biomater..

[118] Feng L., Wu Z., Huang Y., Shen L., Qiao B., Wu J., Wen N., Hu J., Deng B. (2025). Degradation behavior, osteogenesis, and antimicrobial properties of Ga-coated ZK60 Mg alloys for medical implants. J. Magnes. Alloys.

[119] Singh N., Batra U., Kumar K., Ahuja N., Mahapatro A. (2023). Progress in bioactive surface coatings on biodegradable Mg alloys: A critical review towards clinical translation. Bioact. Mater..

[120] Tamay D.G., Gokyer S., Schmidt J., Vladescu A., Huri P.Y., Hasiric V., Hasirci N. (2022). Corrosion Resistance and Cytocompatibility of Magnesium–Calcium Alloys Modified with Zinc- or Gallium-Doped Calcium Phosphate Coatings. ACS Appl. Mater. Interfaces.

[121] Zhang Y., Xu J., Ruan Y.C., Tu M.K., O’Laughlin M., Wise H., Chen D., Tian L., Shi D., Wang J. (2016). Implant-derived magnesium induces local neuronal production of CGRP to improve bone-fracture healing in rats. Nat. Med..

[122] Hou P., Sun Y., Yang W., Wu H., Sun L., Xiu X., Xiu C., Zhang X., Zhang W. (2022). Magnesium promotes osteogenesis via increasing OPN expression and activating CaM/CaMKIV/CREB1 pathway. J. Biomed. Res. B.

[123] Gao J., Feng L., Chen B., Fu B., Zhu M. (2022). The role of rare earth elements in bone tissue engineering scaffolds—A review. Compos. Part B Eng..

[124] Xing Y., Zhong X., Chen S., Wu S., Chen K., Li X., Su M., Liu X., Zhong J., Chen Z. (2023). Optimized osteogenesis of porcine bone-derived xenograft through surface coating of magnesium-doped nanohydroxyapatite. Biomed. Mater..

[125] Zhou W.R., Zheng Y.F., Leeflang M.A., Zhou J. (2013). Mechanical property, biocorrosion and in vitro biocompatibility evaluations of Mg–Li–(Al)–(RE) alloys for future cardiovascular stent application. Acta Biomater..

[126] Ananth K.P., Jayram N.D. (2024). A comprehensive review of 3D printing techniques for biomaterial-based scaffold fabrication in bone tissue engineering. Ann. 3D Print. Med..

[127] de Oliveira M.C.L., Antunes R.A. (2025). Mechanisms and strategies for corrosion control of additively manufactured magnesium alloys. J. Magnes. Alloys.

[128] Badkoobeh F., Mostaan H., Rafiei M., Bakhsheshi-Rad H.R., RamaKrishna S., Chen X. (2023). Additivie manufacturing of biodegradable magnesium-based materials: Design strategies, properties, and biomedical applications. J. Magnes. Alloys.

[129] Jia Q., Gu D. (2014). Selective laser melting additive manufacturing of Inconel 718 superalloy parts: Densification, microstructure and properties. J. Alloys Compd..

[130] Wang W., He L., Yang X., Wang D. (2021). Research on the formation process of selective laser melting Mg-Y-Sm-Zn-Zr alloy. Mater. Sci. Technol..

[131] Munir K., Biesiekierski A., Wen C., Li Y. (2020). 7—Selective laser melting in biomedical manufacturing. Metallic Biomaterials Processing and Medical Device Manufacturing.

[132] Aboulkhair N.T., Simonelli M., Parry L., Ascroft I., Tuck C., Hague R. (2019). 3D printing of Aluminium alloys: Additive Manufacturing of Aluminium alloys using selective laser melting. Prog. Mater. Sci..

[133] Ahmadi M., Bozorgnia Tabary S.A.A., Rahmatabadi D., Ebrahimi M.S., Abrinia K., Hashemi R. (2022). Review of selective laser melting of magnesium alloys: Advantages, microstructure and mechanical characterizations, defects, challenges, and applications. J. Mater. Res. Technol..

[134] Hu D., Wang Y., Zhang D., Hao L., Jiang J., Li Z., Chen Y. (2015). Experimental Investigation on Selective Laser Melting of Bulk Net-Shape Pure Magnesium. Mater. Manuf. Process..

[135] Wei K., Zeng X., Wang Z., Deng J., Liu M., Huang G., Yuan X. (2019). Selective laser melting of Mg-Zn binary alloys: Effects of Zn content on densification behavior, microstructure, and mechanical property. Mater. Sci. Eng. A.

[136] Suchý J., Klakurková L., Man O., Remešová M., Horynová M., Paloušek D., Koutný D., Krištofová P., Vojtěch D., Čelko L. (2021). Corrosion behaviour of WE43 magnesium alloy printed using selective laser melting in simulation body fluid solution. J. Manuf. Process..

[137] Kumar S. (2014). Selective Laser Sintering/Melting. Compr. Mater. Process..

[138] Liu S., Guo H. (2022). Influence of Heat Treatment on Microstructure and Mechanical Properties of AZ61 Magnesium Alloy Prepared by Selective Laser Melting (SLM). Materials.

[139] Lee G.M., Lee J.U., Park S.H. (2021). Effects of post-heat treatment on microstructure, tensile properties, and bending properties of extruded AZ80 alloy. J. Mater. Res. Technol..

[140] Savalani M.M., Pizarro J.M. (2016). Effect of preheat and layer thickness on selective laser melting (SLM) of magnesium. Rapid Prototype J..

[141] Aydin D.S., Bayindir Z., Hoseini M., Pekguleryuz M.O. (2013). The high temperature oxidation and ignition behavior of Mg–Nd alloys part I: The oxidation of dilute alloys. J. Alloys Compd..

[142] Tan Q., Mo N., Jiang B., Pan F., Atrens A., Zhang M.X. (2016). Oxidation resistance of Mg–9Al–1Zn alloys micro-alloyed with Be. Scr. Mater..

[143] Fan J., Chen Z., Yang W., Fang S., Xu B. (2012). Effect of yttrium, calcium and zirconium on ignition-proof principle and mechanical properties of magnesium alloys. J. Rare Earths.

[144] Chang C., Liao H., Yi L., Dai Y., Cox S.C., Yan M., Liu M., Yan X. (2023). Achieving ultra-high strength and ductility in Mg–9Al–1Zn–0.5Mn alloy via selective laser melting. Adv. Powder Mater..

[145] Zhang W., Wang L., Feng Z., Chen Y. (2020). Research progress on selective laser melting (SLM) of magnesium alloys: A review. Optik.

[146] Yu L., Nakata K., Yamamoto N., Liao J. (2009). Texture and its effect on mechanical properties in fiber laser weld of a fine-grained Mg alloy. Mater. Lett..

[147] Teng H., Li T., Zhang X., Zhang Z. (2008). Influence of sub-rapid solidification on microstructure and mechanical properties of AZ61A magnesium alloy. Trans. Nonferrous Met. Soc. China.

[148] Sezer N., Evis Z., Koç M. (2021). Additive manufacturing of biodegradable magnesium implants and scaffolds: Review of the recent advances and research trends. J. Magnes. Alloys.

[149] Kopp A., Derra T., Müther M., Jauer L., Schleifenbaum J.H., Voshage M., Jung O., Smeets R., Kröger N. (2019). Influence of design and postprocessing parameters on the degradation behavior and mechanical properties of additively manufactured magnesium scaffolds. Acta Biomater..

[150] Qin Y., Wen P., Guo H., Xian D., Zheng Y., Jauer L., Poprawe R., Voshage M., Schleifenbaum J.H. (2019). Additive manufacturing of biodegradable metals: Current research status and future perspectives. Acta Biomater..

[151] Chen C., Sun G., Du W., Liu J., Zhang H. (2023). Effect of equivalent heat input on WAAM Al-Si alloy. Int. J. Mech. Sci..

[152] Gu D., Shi X., Poprawe R., Bourell D.L., Setchi R., Zhu J. (2021). Material-structure-performance integrated laser-metal additive manufacturing. Science.

[153] Marques D.A., Oliviera J.P., Baptista A.C. (2023). A Short Review on the Corrosion Behaviour of Wire and Arc Additive Manufactured Materials. Metals.

[154] Shah A., Aliyev R.A., Zeidler H., Krinke S. (2023). A Review of the Recent Developments and Challenges in Wire Arc Additive Manufacturing (WAAM) Process. J. Manuf. Mater. Process..

[155] Siddiqui N.A., Muzamil M., Jamil T., Hussain G. (2025). Heat sources in wire arc additive manufacturing and their impact on macro-microstructural characteristics and mechanical properties—An overview. Smart Mater. Manuf..

[156] Chen T., Hu S., Li S., Huo Q. (2022). Uncovering the unexpected changes of creep properties in AZ-series Mg alloys. Mater. Sci. Eng. A.

[157] Tolouie E., Jamaati R. (2018). Effect of β–Mg17Al12 phase on microstructure, texture and mechanical properties of AZ91 alloy processed by asymmetric hot rolling. Mater. Sci. Eng. A.

[158] Li J., Qiu Y., Yang J., Sheng Y., Yi Y., Zeng X., Chen L., Yin F., Su J., Zhang T. (2023). Effect of grain refinement induced by wire and arc additive manufacture (WAAM) on the corrosion behaviors of AZ31 magnesium alloy in NaCl solution. J. Magnes. Alloys.

[159] Li J., Huang M., Hou J., Yang Y., Xu G., Mo N., Tang W., Shi Y. (2024). Effect of heat treatment on the microstructure and corrosion properties of plasma wire arc additive manufactured Mg-Gd-Y-Zr alloy. Mater. Commun..

[160] Cherru J.A., Davies H.M., Mehmood D., Lavery N.P., Brown S.G.R., Sienz J. (2014). Investigation into the effect of process parameters on microstructural and physical properties of 316L stainless steel parts by selective laser melting. Int. J. Adv. Manuf. Technol..

[161] Yadroitsev I., Bertrand P., Smurov I. (2007). Parametric analysis of the selective laser melting process. Appl. Surf. Sci..

[162] Zhang C., Li Z., Zhang J., Tang H., Wang H. (2023). Additive manufacturing of magnesium matrix composites: Comprehensive review of recent progress and research perspectives. J. Magnes. Alloys.

[163] Mirkoohi E., Li D., Garmestani H., Liang S.Y. (2021). Residual stress modeling considering microstructure evolution in metal additive manufacturing. J. Manuf. Process..

[164] Srivastava S., Garg R.K., Anish S., Sharma V.S. (2020). Measurement and Mitigation of Residual Stress in Wire-Arc Additive Manufacturing: A Review of Macro-Scale Continuum Modelling Approach. Arch. Comput. Methods Eng..

[165] Yu W.H., Sing S.L., Chua C.K., Kuo C.N., Tian X.L. (2019). Particle-reinforced metal matrix nanocomposites fabricated by selective laser melting: A state-of-the-art review. Prog. Mater. Sci..

[166] Peng X., Kong L., Fuh J.Y.H., Wang H. (2021). A Review of Post-Processing Technologies in Additive Manufacturing. J. Manuf. Mater. Process..

[167] Kumar L.G.S., Thirumalaikumarasamy D., Krthikelyan K., Mathanbabu M., Ashokkumar M., Ramachandran C.S. (2023). An overview of recent trends and challenges of post treatments on magnesium alloys. Mater. Today Proc..

[168] Chenguang H., Changjun C., Min Z. (2021). Effects of Ag content and heat treatment on the microstructure and properties of SLMed AZ61 Mg–Al–Zn alloy. Appl. Phys. A Mater. Sci. Process..

[169] Zhang Z., Wang L., Zhang R., Yin D., Zhao Z., Bai P., Liu B., Wang F. (2022). Effect of solution annealing on microstructures and corrosion behavior of wire and arc additive manufactured AZ91 magnesium alloy in sodium chloride solution. J. Mater. Res. Technol..

[170] Mostafei A., Zhao C., He Y., Ghiaasiaan S.R., Shi B., Shao S., Shamasaei N., Wu Z., Kouraytem N., Sun T. (2022). Defects and anomalies in powder bed fusion metal additive manufacturing. Curr. Opin. Solid State Mater. Sci..

[171] Yan X., Lupoi R., Wu H., Ma W., Liu M., O’Donnell G., Yin S. (2019). Effect of hot isostatic pressing (HIP) treatment on the compressive properties of Ti6Al4V lattice structure fabricated by selective laser melting. Mater. Lett..

[172] Cegan T., Pagac M., Jurica J., Skotnicova K., Hajnys J., Horsak L., Soucek K., Krpec P. (2020). Effect of Hot Isostatic Pressing on Porosity and Mechanical Properties of 316 L Stainless Steel Prepared by the Selective Laser Melting Method. Materials.

[173] Tradowsky U., White J., Ward R.M., Read N., Reimers W., Attallah M.M. (2016). Selective Laser Melting of AlSi10Mg: Influence of Post-Processing on the Microstructural and Tensile Properties Development. Mater. Des..

[174] Masuo H., Tanaka Y., Morokoshi S., Yagura H., Uchida T., Yamamoto Y., Murakami Y. (2018). Influence of defects, surface roughness and HIP on the fatigue strength of Ti-6Al-4V manufactured by additive manufacturing. Int. J. Fatigue.

[175] Goel S., Sittiho A., Charit I., Klement U., Joshi S.V. (2019). Effect of Post-Treatments under Hot Isostatic Pressure on Microstructural Characteristics of EBM-built Alloy 718. Addit. Manuf..

[176] Gangireddy S., Gwalani B., Liu C., Faierson E.J., Mishra R.S. (2019). Microstructure and mechanical behavior of an additive manufactured (AM) WE43-Mg alloy. Addit. Manuf..

[177] Tan Q., Zhang J., Sun J., Fan Z., Li G., Yin Y., Liu Y., Zhang M.X. (2020). Inoculation Treatment of an Additively Manufactured 2024 Aluminium Alloy with Titanium Nanoparticles. Acta Mater..

[178] Majeed T., Siddiquee A.N., Mehta Y., Ansari N., Alam M.N., Lone N.F. (2022). A comparative study on friction stir welding of tailor weld blanks. Mater. Sci. Technol..

[179] Li B., Hou X., Teng B. (2019). Effects of friction stir process and subsequent aging treatment on the microstructure evolution and mechanical properties of Mg-Gd-Y-Zn-Zr alloy. Mater. Charact..

[180] Macias J.G.S., Elangeswaran C., Lv Z., Hooreweder B.V., Adrien J., Marie E., Buffiere J.Y. (2019). Ductilisation and fatigue life enhancement of selective laser melted AlSi10Mg by friction stir processing. Scr. Mater..

[181] Yang G., Zhang W.Q., Zhang J., Yi J.Z., Cui Y.F. (2023). Evolution of microstructure of WE43 magnesium alloys fabricated by laser deposition manufacturing with subsequent friction stir processing. Mater. Lett..

[182] Wei J., He C., Qie M., Li Y., Tian N., Qin G., Zuo L. (2022). Achieving high performance of wire arc additive manufactured Mg–Y–Nd alloy assisted by interlayer friction stir processing. J. Mater. Process. Technol..

[183] Manam N.S., Harun W.S.W., Shri D.N.A., Ghani S.A.C., Kurniawan T., Ismail M.H., Ibrahim M.H.I. (2017). Study of corrosion in biocompatible metals for implants: A review. J. Alloys Compd..

[184] Herber V., Okutan B., Antonoglou G., Sommer N.G., Payer M. (2021). Bioresorbable Magnesium-Based Alloys as Novel Biomaterials in Oral Bone Regeneration: General Review and Clinical Perspectives. J. Clin. Med..

[185] Ren J., Jiang Z., He J., Wang X., Jin W., Yu Z. (2025). Current status and perspectives on design, fabrication, surface modification, and clinical applications of biodegradable magnesium alloys. J. Magnes. Alloys.

[186] Romanos G.E., Delgardo-Ruiz R.A., Monero G., López-López P.J., de Val J.E.M.S., Calvo-Guirado C. (2015). Role of mechanical compression on bone regeneration around a particulate bone graft material: An experimental study in rabbit calvaria. Clin. Oral Implant. Res..

[187] Manescu (Paltanea) V., Antoniac I., Antoniac A., Laptoiu D., Paltanea G., Ciocoiu R., Nemoianu I.V., Gruionu L.G., Dura H. (2023). Bone Regeneration Induced by Patient-Adapted Mg Alloy-Based Scaffolds for Bone Defects: Present and Future Perspectives. Biomimetics.

[188] Nyman S., Lindhe J., Karring T., Rylander H. (1982). New attachment following surgical treatment of human periodontal disease. J. Clin. Periodontol..

[189] Deepak J.R., Joy N., Arukumar T., Srivatsan R., Gnanasekar R. (2021). Tribological investigation of magnesium rare earth alloy for orthopedic application. Mater. Proc..

[190] Mostaed E., Sikora-Jasinska M., Drelich J.W., Vedani M. (2018). Zinc-based alloys for degradable vascular stent applications. Acta Biomater..

[191] Chen C., Chen J., Wu W., Shi Y., Jin L., Petrini L., Shen L., Yuan G., Ding W., Ge J. (2019). In vivo and in vitro evaluation of a biodegradable magnesium vascular stent designed by shape optimization strategy. Biomaterials.

[192] Zhou X., Fang Y., Zhang Y., Xiong Z. (2024). Retrospective: Advances and Opportunities of 3D Bioprinting in China over Three Decades. Addit. Manuf. Front..

[193] Zolfagharian A., Kaynak A., Bodaghi M., Kouzani A.Z., Gharaie S., Nahavandi S. (2020). Control-Based 4D Printing: Adaptive 4D-Printed Systems. Appl. Sci..

[194] Taltavull C., Torres B., López A.J., Rodrigo P., Otero E., Rams J. (2012). Selective laser surface melting of a magnesium-aluminium alloy. Mater. Lett..

[195] Kaushik, Kumar N., Kumar S.S., Margabandu V. (2022). Magnesium role in additive manufacturing of biomedical implants—Challenges and opportunities. Addit. Manuf..

[196] Wang Y., Fu P., Wang N., Peng L., Kang B., Zeng H., Yuan G., Ding W. (2020). Challenges and Solutions for the Additive Manufacturing of Biodegradable Magnesium Implants. Engineering.

